# BI 2536 induces gasdermin E-dependent pyroptosis in ovarian cancer

**DOI:** 10.3389/fonc.2022.963928

**Published:** 2022-08-09

**Authors:** Jianting Huo, Yuhong Shen, Yuchen Zhang, Lifei Shen

**Affiliations:** ^1^ Department of General Surgery, Ruijin Hospital, Shanghai Jiaotong University School of Medicine, Shanghai, China; ^2^ Shanghai Minimally Invasive Surgery Center, Ruijin Hospital, Shanghai Jiaotong University School of Medicine, Shanghai, China; ^3^ Department of Obstetrics and Gynecology, Ruijin Hospital, Shanghai Jiaotong University School of Medicine, Shanghai, China

**Keywords:** pyroptosis, apoptosis, ovarian cancer, small molecule drug, immunotherapy

## Abstract

**Background:**

The frequent emergence of drug resistance to chemotherapy is a major obstacle for the treatment of ovarian cancer. There is a need for novel drugs to fulfill this challenge. Pyroptosis-inducing drugs can inhibit tumor growth. However, their roles in ovarian cancer have not been demonstrated.

**Methods:**

We tested the effectiveness of a novel drug, BI 2536, which we found in colorectal cancer. Cell proliferation, cell cycle, and drug-induced apoptosis and pyroptosis were tested. *In vivo* treatments were performed using a cell-derived xenograft model.

**Results:**

BI 2536 significantly inhibited the proliferation of ovarian cancer cells and induced cell cycle arrest at the G2/M phases. After BI 2536 treatment, DNA fragmentation and PS exposure on the outside of apoptotic cells were detected. Moreover, the pyroptotic phenotype of ovarian cancer cells along with the release of LDH and HMGB1 were observed, indicating the leakage of cells. Western blot analysis verified that BI 2536 induced GSDME-mediated pyroptosis. Pyroptosis was abolished after additional treatment with Z-DEVD-FMK, a caspase-3 inhibitor. Thus, BI 2536 induced pyroptosis in ovarian cancer through the caspase-3/GSDME pathway. *In vivo* experiments further demonstrated the antitumoral effect and ability of BI 2536 to accumulate CD8^+^ T cells in ovarian cancer.

**Conclusion:**

In this study, we identified BI 2536 as an effective anti-ovarian cancer drug that inhibits proliferation, arrests the cell cycle, induces apoptosis and pyroptosis, and leads to the accumulation of CD8^+^ T cells in tumor sites. Drug-induced pyroptosis may have promising prospects for reducing side effects and activating immune responses.

## Introduction

The mortality-to-incidence ratio in ovarian cancer is over 0.6, and studies estimated that 1 in 6 women die within the first three months of diagnosis (1). The potentially high morbidity and mortality are partially due to late-stage diagnoses and resistance to previous treatments (2). Cytoreductive surgery and platinum-based chemotherapy remain the main therapies. Although research regarding the treatment of ovarian cancer has progressed rapidly, the majority of women presenting with advanced stages seldom benefit from these treatments. Therefore, finding effective therapeutic agents to inhibit the occurrence of drug resistance is important to prolong the survival rate of patients.

The evasion of tumor cell death, especially apoptosis, accounts for metastasis, occurrence, and chemotherapy resistance (3, 4). Thus, current studies have put effort into inducing nonapoptotic cell death, which is shown to be an effective approach to improving therapeutic efficacies. Nonapoptotic cell death modes mainly include autophagy, pyroptosis, ferroptosis, and necroptosis. Pyroptosis is ascribed to the proteolytic fragmentation of gasdermin D (GSDMD) by caspase-1/4/5 (5, 6) and is verified to function through caspase-3 cleavage of gasdermin E (GSMDE) (7, 8). Pyroptotic cells undergo plasma membrane leakage after swelling, and many bubble-like protrusions appear on the surface of the cellular membrane (9). They allow immunogenic components, including damage-associated molecular patterns (DAMPs), such as high mobility group proteins B1 (HMGB1) and inflammatory cytokines (i.e., interleukin [IL]-1β), to be released into the tumor microenvironment (10). Recently, the role of pyroptosis-inducing drugs in cancer treatments has attracted increasing attention. Zhou B et al. (11) demonstrated that iron-activated ROS induce pyroptosis *via* a Tom20/Bax/caspase/GSDME pathway, which plays a role in melanoma therapy. Yu J et al. (12) reported that lobaplatin-induced GSDME-mediated pyroptosis downstream of the ROS/JNK/Bax pathway and caspase-3/-9 activation inhibits the growth of colorectal cancer cells. Since drug-induced pyroptosis has not been reported in ovarian cancer, effective drugs able to trigger pyroptosis could provide an option for ovarian cancer treatment.

Previously, we found a novel small molecule inhibitor, BI 2536, which can induce GSDME-mediated pyroptosis in colorectal cancer (13). Since there are few studies on the role of pyroptosis in ovarian cancer, we wondered whether BI 2536 could have a similar antitumor effect in ovarian cancer. In this study, the antiproliferative and cell cycle arrest roles of BI 2536 were verified in ovarian cancer. We examined the mechanism of regulated cell death and surprisingly found that BI 2536 can trigger GSDME-dependent pyroptosis, concurrent with caspase-3-mediated apoptosis. *In vivo* experiments further confirmed the therapeutic function of BI 2536 in ovarian cancer mouse models. Moreover, BI 2536 increased CD8^+^ T cell infiltration in tumor sites. As a result, our study found a promising agent for ovarian cancer and illustrated that it can induce pyroptosis through the caspase-3/GSDME pathway, which may have reasonable prospects in overcoming the drug resistance of ovarian cancer and improving the outcomes of these patients.

## Materials and methods

### Cell culture and reagents

Human ovarian cell line A2780 cell was purchased from the American Type Culture Collection (ATCC, USA). It was cultured in RPMI 1640 medium supplemented with 10% FBS and 1% penicillin/streptomycin in a humidified incubator at 37°C in 5% CO2. Small-molecule inhibitor, BI 2536, was purchased from TargetMol (China), dissolved in DMSO at a storage concentration of 10mM.

### Cell viability assay

Cell survival rates were estimated by the Cell Counting Kit (CCK)-8 assay (Beyotime Biotec). Approximately 8,000 cells were seeded in 96-well plates with 100 μl of medium in each well. After 24 hours, drugs were added for another three-day treatment. Each well was incubated with 10 μl CCK-8 solution for two hours at 37 °C in the dark, and the absorbance at 450 nm was measured by a microplate spectrophotometer (Tecan).

### Colony formation assay

Ovarian cancer cells were seeded in a 12-well plate at concentration of 500 cells/well before treated with indicated drugs (DMSO, 0.1 μM, 0.5 μM, 1.0 μM, 2.0 μM BI 2536). After visible cloning appeared, the cell medium was discarded, and the colonies were fixed with 4% formaldehyde for 30 min and later stained with crystal violet solution for 10 min. The stained colonies were photographed after PBS washing.

### Cell cycle assessment

Cells were seeded in a 12-well plate and treated with indicated drugs (DMSO, 0.1 μM, 0.5 μM, 1.0 μM, 2.0 μM BI 2536) for three days. Then cells were washed with PBS, harvested, and fixed with anhydrous ethanol overnight. After washed with PBS, PI/RNase staining (550825, BD Biosciences) was added to suspend cells and incubate for 30 minutes in dark. The cell cycle distribution was determined by flow cytometry (BD Biosciences).

### EdU proliferation assay

Cells were seeded in a 12-well plate. After the treatment with indicated drugs (DMSO, 0.5 μM BI 2536) for three days, EdU (Beyotime Biotec) was added at a working concentration of 10μM for 2 hours. The cells were washed with PBS, fixed with 4% formaldehyde for 30 min, permeabilized with 0.25% Triton X-100 (Sigma), and incubated with Click Additive Solution (Beyotime Biotec) according to the manufacturer’s instructions, following by staining with Hoechst (Beyotime Biotec). After the staining, the cells were observed using a fluorescent microscope (Olympus).

### Apoptosis assay

After treatment with drugs (0.1 μM, 0.5 μM, 1.0 μM, 2.0 μM BI 2536) or the DMSO control for three days, the cells were stained with Annexin-V-APC (550474, BD Biosciences) and propidium iodide (PI; 556463, BD Biosciences) according to the manufacturer’s protocol and assessed with a flow cytometer (BD Biosciences). Annexin-V^+^ PI^-^ cells were classified as early apoptotic cells, and Annexin-V^+^ PI^+^ cells were classified as late apoptotic cells.

### TdT-mediated dUTP Nick-End Labeling (TUNEL) assay

The DMSO- and 0.5 μM BI 2536-treated cells were labelled with FITC using the One Step TUNEL Apoptosis Assay Kit (Meilunbio) according to the manufacturer’s recommendations. The nucleus was stained with 4′,6-diamidino-2-phenylindole (DAPI; Beyotime Biotec). Immunofluorescent staining was observed using a fluorescent microscope (Olympus).

### Western blot analysis

Cells were lysed in RIPA buffer (Solarbio) containing phenylmethylsulfonyl fluoride (PMSF; Sigma-Aldrich) protease inhibitor, and the protein concentration was measured with the Pierce™ BCA Protein Assay Kit (ThermoFisher Scientific). Protein extracts (20 μg) were subjected to sodium dodecyl sulfate polyacrylamide gel electrophoresis (SDS-PAGE), then transferred onto polyvinyl difluoride (PVDF) membranes (Millipore) and blocked with 5% BSA for one hour at room temperature. The membranes were incubated with primary antibodies targeting Bcl-2 (60178-1-Ig, Proteintech), cIAP-1 (ab108361, Abcam), GSDME (A7432, ABclonal), caspase-3 (ab32351, Abcam; 1:1000 dilution) and GAPDH (60004-1-Ig, Proteintech, 1:5000 dilution) at 4 °C overnight. After washing three times with Tris Buffered Saline with Tween (TBST) buffer, the membranes were incubated with horseradish peroxidase-conjugated secondary antibodies at room temperature for one hour. Western horseradish peroxidase (HRP) Substrate (Millipore) was added to visualize protein bands.

### LDH and HMGB1 release assay

The cells were pretreated with the respective drugs (DMSO, 0.1 μM, 0.5 μM, 1.0 μM, 2.0 μM BI 2536) for three days. The LDH release was measured with the LDH Release Assay Kit (Beyotime Biotec) according to the manufacturer’s instructions. In brief, 120 μl of supernatant was transferred to a 96-well plate, and the response mixture was added and incubated in the dark for 30 minutes at room temperature. The absorbance value at 490 nm was then measured, referenced by 1000 nm. HMGB1 levels in the supernatant medium of the cells were determined by an enzyme linked immunosorbent assay (ELISA) according to the manufacturer’s instructions. HMGB1 (Human) Matched Antibody Pair was purchased from Abnova (H00003146-AP41, Taiwan, China) and the human HMGB1 ELISA kit was purchased from Senxiong BioTech (SX01187, Shanghai, China).

### Cell-derived xenograft model

All animal studies were approved by the Biomedical Ethics Committee of Ruijin Hospital. Tumor cells (3×10^6^) in the logarithmic growth phase were subcutaneously injected into the flanks of 4-week-old male BALB/c nude mice. One week after injection, the mice were randomly divided into two groups of three, and respectively treated with a vehicle control and BI 2536 (10 mg/kg) *via* intraperitoneal injection. When four doses of drugs were injected or the diameter of tumors reached twenty millimeters, mice were sacrificed. Tumor volumes were calculated as length × width^2^ × 0.5.

### Hematoxylin-eosin (H&E) and immunohistochemistry (IHC) staining

For H&E staining, tissue samples were fixed in 4% paraformaldehyde, washed with PBS and transferred to 70% ethanol. The samples were then embedded in paraffin, sectioned and stained with H&E. For IHC staining, paraffin-embedded tissues were deparaffinized in xylene, passed through graded alcohols and the antigen was retrieved with citrate buffer in a steam pressure cooker. The samples were then incubated with anti-Ki67 (ab15580, Abcam) or anti-CD8 (ab217344, Abcam), washed in PBS, and incubated with horseradish peroxidase-conjugated secondary antibody. Slides were counterstained with hematoxylin, dehydrated in graded alcohol and xylene, and coverslipped with mounting solution. The number of positive cells were counted in each high-power field (HPF) by two independent pathologists.

### Statistical analysis

Statistical analysis was performed with GraphPad Prism 7.0 (GraphPad Software). The statistical significance between two groups was analyzed by the Student’s *t* test. All tests were two-tailed, and *P*-values < 0.05 were considered significant (* *P* < 0.05, ** *P* < 0.01, and *** *P* < 0.001). Relative protein expression was analyzed by ImageJ software.

## Results

### BI 2536 inhibits ovarian cancer cells growth *in vitro*

**Figure 1:**
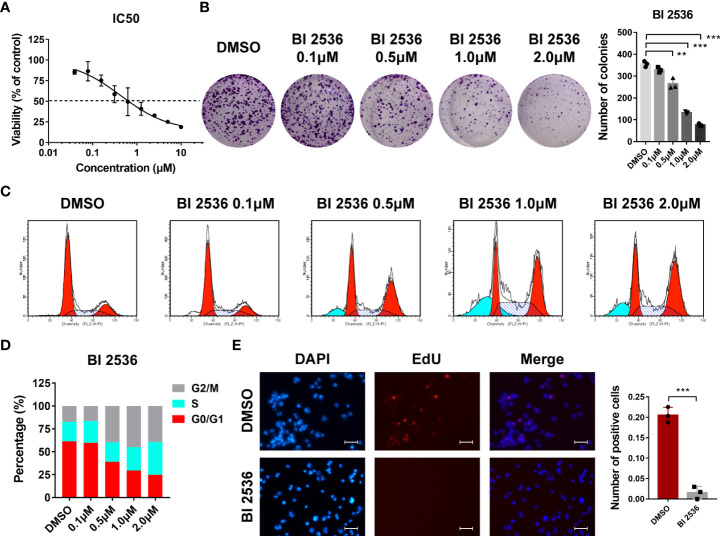
BI 2536 inhibits ovarian cancer cells growth *in vitro* **(A)** Half-maximal inhibitory concentration curves of BI 2536 evaluated by CCK-8 assay. **(B)** Colony formation assay of A2780 treated with the indicated concentration of BI 2536. **(C, D)** Flow cytometry analysis of the proportion of different cell cycle phases in treated A2780 cells. **(E)** Representative images showing fluorescence staining of BI 2536-treated cells using EdU proliferation assay. **P < 0.01, and ***P < 0.001.

We first evaluated the dose-response relationship of BI 2536 in an ovarian cancer cell line A2780. BI 2536 significantly inhibited the viability of A2780 with an IC50 of 0.439μM (**Figure 1A**). In colony formation assay, the number of colonies reduced after the treatment with BI 2536 (**Figure 1B**), revealing that BI 2536 inhibited cell proliferation in a dose dependent manner. Cell cycle assessment indicated that A2780 treated with BI 2536 underwent an increased proportion of G2/M phases and a reduced proportion of G0/G1 phases (**Figures 1C, D**). The EdU proliferation assay showed that, when treated with 0.5μM BI 2536, ovarian cancer cells at proliferation phases were decreased (**Figure 1E**). Taken together, these results emphasized that BI 2536 inhibited the proliferation of ovarian cancer cells and induced cell cycle arrest at the G2/M phases.

### BI 2536 induces apoptosis of ovarian cancer cells

**Figure 2:**
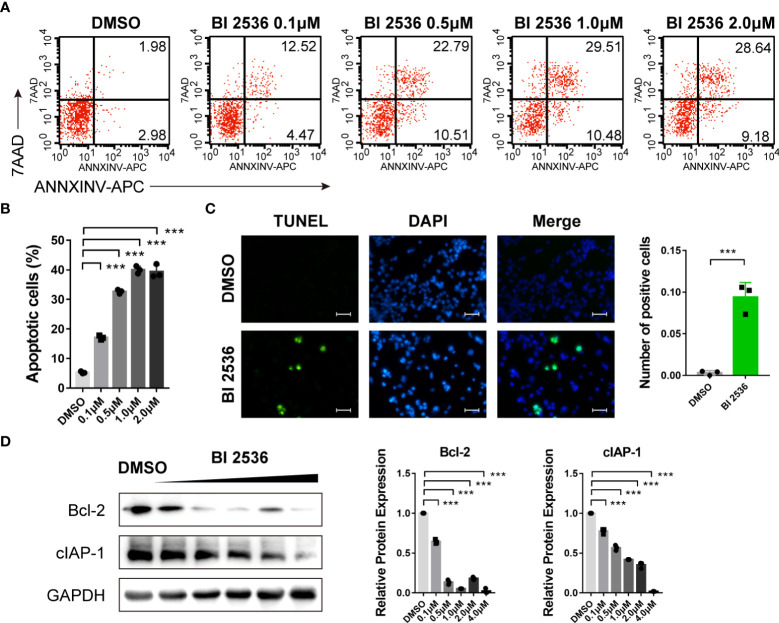
BI 2536 induces apoptosis of ovarian cancer cells **(A, B)** Flow cytometry analysis of Annexin V-APC and PI staining of apoptotic cells. **(C)** TUNEL assay of A2780 cells treated with BI 2536 or DMSO. **(D)** Immunoblotting analysis of the indicated proteins extracted from BI 2536-treated A2780 cells. Scale bar = 200μm. ***P < 0.001.

Since hypodiploid peak appeared before G0/G1 peak in the cell cycle analysis (**Figure 1C**), which is a sign of early apoptosis of cells, we then investigated the role of BI 2536 in apoptosis. Flow cytometric analysis revealed that the percentage of Annexin V positive and/or PI positive A2780 cells increased significantly after treated with BI 2536 (**Figures 2A, B**). DNA fragmentation stained using the TUNEL assay confirmed apoptosis induction of BI 2536 (**Figure 2C**). We then examined the apoptosis-related protein level alteration by the western blot assay. The expressions of Bcl-2 and cIAP-1 were significantly downregulated after A2780 cells were treated with BI 2536 (**Figure 2D**). These data suggested that BI 2536 induced apoptosis of ovarian cancer cells.

### BI 2536 induces GSDME-dependent pyroptosis of ovarian cancer cells

**Figure 3:**
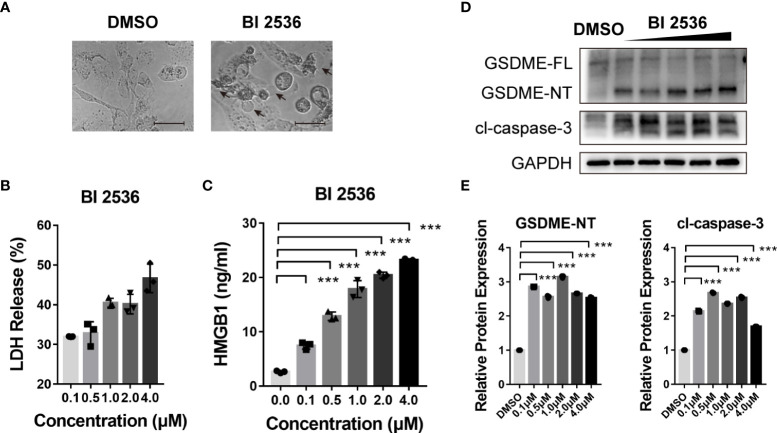
BI 2536 induces GSDME-dependent pyroptosis of ovarian cancer cells **(A)** Representative images of A2780 cells treated with DMSO and BI 2536 for 72 hours. Pyroptotic cell morphology is pinpointed by arrows. **(B)** LDH and **(C)** HMGB1 release from A2780 cells treated with BI 2536. Each column represents the mean value of three biological replicates, and error bars indicate SD. **(D, E)** Immunoblotting analysis of the indicated proteins extracted from BI 2536-treated cells. Scale bar = 200μm. ***P < 0.001.

We previously found BI 2536 induced concurrent apoptosis and pyroptosis in colorectal cancer cells (13). Here, we observed the morphology of BI 2536-treated A2780 cells. These cells were swelling and exhibited large bubbles of the plasma membrane (**Figure 3A**). The level of LDH release was elevated in a dose dependent manner (**Figure 3B**). Moreover, the release level of HMGB1, one of the DAMPs, was increased simultaneously (**Figure 3C**), indicating cell membrane rupture and leakage. Cells undergoing necroptosis have a similar swelling phenotype and change in membrane permeability (14). To distinguish the pathway by which BI 2536 induced cell death, we examined the protein level alteration by the western blot assay. Cleavage of caspase-3 was observed. Meanwhile, GSDME was cleaved to generate N-terminal fragments (a marker of pyroptosis, **Figures 3D, E**). In summary, these results indicated that BI 2536-induced apoptosis cooccurred with GSDME-mediated pyroptosis.

### Caspase-3 inhibition eliminates BI 2536-induced pyroptosis of ovarian cancer cells

**Figure 4:**
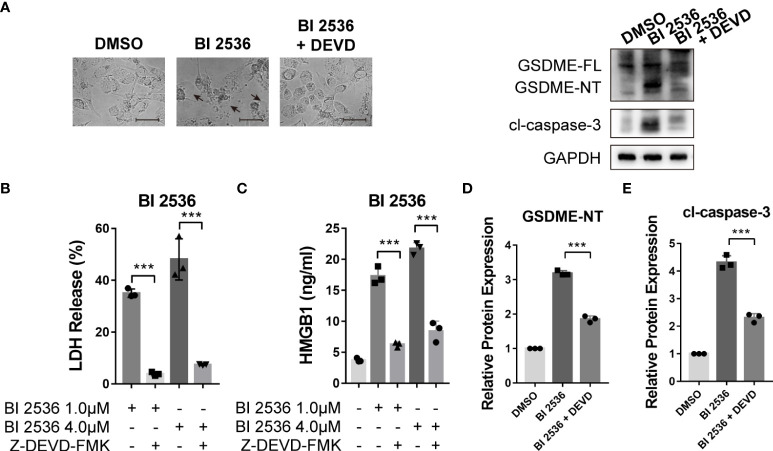
Caspase-3 inhibition eliminates BI 2536-induced pyroptosis of ovarian cancer cells **(A)** Bright-field microscopy images of BI 2536-treated A2780 cells. Altered morphology was shown when treated with caspase-3 inhibitor Z-DEVD-FMK. **(B)** LDH and **(C)** HMGB1 release from A2780 cells treated with BI 2536. Each column represents the mean value of three biological replicates, and error bars indicate SD. The level of LDH and HMGB1 was lowered when adding DEVD. **(D, E)** Immunoblotting analysis of the indicated proteins extracted from BI 2536-treated cells. BI 2536-induced cleavages of GSDME and caspase-3 were inhibited by DEVD. Scale bar = 200μm. ***P < 0.001.

Since GSDME-mediated pyroptosis has emerged as a new cancer treatment strategy (15–17), we focused on the mechanism of BI 2536-induced pyroptosis. BI 2536 was shown to induce pyroptosis triggered by GSDME cleavage (**Figure 3D**). As caspase-3 was cleaved concurrently, we speculated that GSDME-mediated pyroptosis was dependent on caspase-3 as previously reported (8). To verify the function of caspase-3 in pyroptosis, we employed caspase-3 inhibitor, Z-DEVD-FMK (DEVD), to restrain the cleavage of caspase-3. We found that BI 2536-induced cell swelling was abolished by DEVD (**Figure 4A**). The release of LDH and HMGB1 was remarkably reduced in presence of DEVD (**Figures 4B, C**). In the molecular level, the inhibition of caspase-3 suppressed BI 2536-induced generation of N-terminal fragments of GSDME (**Figures 4D, E**), indicating that pyroptosis was secondary to apoptosis. In summary, these results indicated that GSDME-mediated pyroptosis triggered by BI 2536 was due to the activation of caspase-3.

### BI 2536 inhibits ovarian cancer cells growth and accumulate CD8^+^ T cells *in vivo*

**Figure 5:**
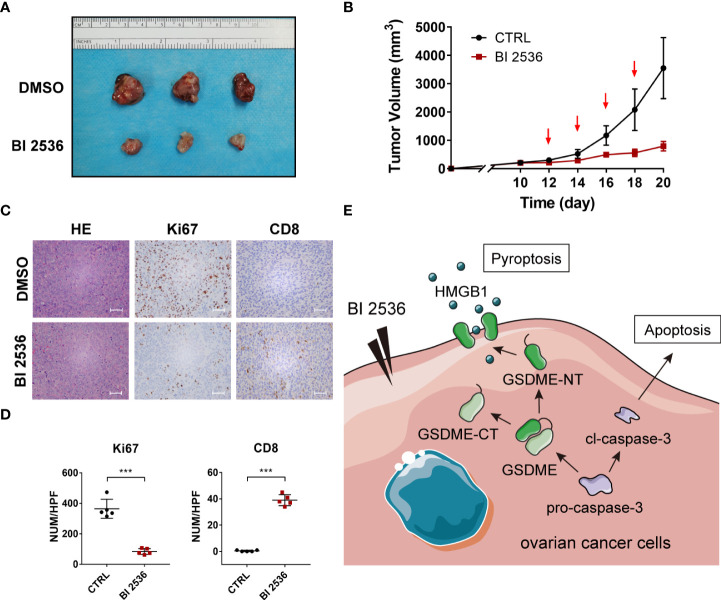
BI 2536 inhibits ovarian cancer cells growth *in vivo* **(A)** Images and **(B)** tumor growth curves of xenograft tumors for DMSO and BI 2536 treatment among BALB/c-nude mice. **(C)** H&E and IHC staining of drug-treated subcutaneous tumors. **(D)** The number of Ki 67 and CD8 positive cells in each high-power field (HPF). **(E)** A schematic representation of proposed mechanism for GSDME-dependent pyroptosis induced by BI 2536 in ovarian cancer cells. Scale bar = 200μm. ***P < 0.001.

We validated the drug response *in vivo* using a subcutaneous tumor model. One-week post-injection, mice were treated with DMSO and BI 2536 *via* intraperitoneal injection every other day four times. After the treatment, the growth rates of the tumors were robustly inhibited compared with the vehicle control values (**Figures 5A, B**). IHC staining of Ki67 revealed that cell proliferation was significantly reduced when treated with BI 2536 (**Figures 5C, D**). Moreover, more CD8^+^ cells were observed in BI 2536-treated tumors (**Figures 5C, D**). Hence, we concluded that BI 2536 inhibited ovarian cancer cells growth *in vivo*.

## Discussion

Although tumors can be alleviated to an undetectable level using current chemotherapy, they often recur at primary or distant sites if residual cancer cells are present after initial therapy (18–20). Lack of effective treatment is one of the major causes of death from ovarian cancer among women with malignant gynecological tumors (21). Cancer treatments based on molecular targeted therapy are promising for improving therapeutic efficacy by inhibiting uncontrolled cancer cell proliferation and inducing apoptosis.

In this research, we found that a small molecule drug, BI 2536, has great efficacy in inhibiting ovarian cancer growth and proliferation. In particular, BI 2536 induced concurrent apoptosis (**Figure 2**) and pyroptosis (**Figure 3**) in ovarian cancer cells. Pyroptosis, as a form of immunogenic cell death, is a potential tumor treatment strategy (8, 9, 16, 22, 23). Several promising small molecules and nanomaterials have been reported in different cancers, such as non-small cell lung carcinoma (24–26), colorectal cancer (12, 27–29), hepatocellular carcinoma (30–32), breast cancer (33–36), melanoma (11, 16, 34), and glioblastoma (37–40). However, there have been only two studies on pyroptosis-induced therapy in ovarian cancer. Zhang R et al. (41) reported that nobiletin, a food-derived phytochemical extracted from citrus fruits, contributed to GSDMD- or GSDME-mediated pyroptosis. Liang J et al. (42) demonstrated that osthole triggers GSDME-dependent pyroptosis in ovarian cancer cells. However, they did not verify the mechanism of pyroptosis clearly.

BI 2536, a Plk1 enzyme inhibitor, was suggested to induce mitotic arrest and a subsequent surge in apoptosis (43) and suppress the expression of epithelial-mesenchymal transition markers and 3D spheroid formation in breast cancer (44). The Plk1 kinase inhibitor BI 2536 was previously confirmed to induce pyroptosis in the caspase-3/GSDME pathway in esophageal squamous cell carcinoma (45). However, no current research has shown the pyroptosis-induced role of BI 2536 in ovarian cancer. Our results demonstrated that BI 2536 can induce GSDME-dependent pyroptosis concurrent with caspase-3-mediated apoptosis. Moreover, the cleavage of GSDME is dependent on the activation of caspase-3 (**Figure 4**). When cells were treated with both BI 2536 and caspase-3 inhibitor, the HMGB1 release and the expression of GSDME-NT and cleaved caspase-3 were higher than those of cells only treated with DMSO (**Figures 4C, E**). Therefore, proteins other than caspase-3 might also promote the cleavage of the linker region between the C and N terminals of GSDME. We speculated that the terminal form of cell death after caspase-3 activation may be determined by the drug efficacy and GSDME level, and emphasized that different types of cell death operating synergistically, not mutually exclusively, could contribute to the improvement of the toxic effects of treatment. The safety and effectiveness of the drug were tested *in vivo* (**Figure 5**). Combined therapy with traditional chemotherapy may be expected to have a synergistic effect to improve the prognosis of patients.

GSDME-mediated pyroptosis induced by BI 2536 was shown to be antitumorigenic in ovarian cancer. BI 2536 also showed its role in the accumulation of CD8^+^ T cells in tumor sites (**Figure 5**). BI 2536-induced immune cell accumulation may have great translational prospects in improving the effect of immunotherapy. However, one recent study showed that GSDME expression at the mRNA level is decreased in ovarian tumors compared to healthy tumors (46). Thus, ovarian cancer cells may evade GSMDE-mediated pyroptotic cell death. Further research can be performed to identify a method to upregulate the expression of GSDME and improve the antitumorigenic function of BI 2536. Peng Z et al. (24) reported that chemokines, such as MIP-1α, MIP-1β, MIP-2, and IP-10, were increasingly released in tumor tissue after drug treatment and have been shown to play an important role in T cell recruitment. The mechanism of pyroptosis in the accumulation of immune cells needs further research.

In conclusion, the small molecule drug BI 2536 was verified to inhibit the proliferation of ovarian cancer cells and induce concurrent apoptosis and pyroptosis through the caspase-3/GSDME pathway, and it showed both *in vitro* and *in vivo* antitumoral activity and the ability to accumulate CD8^+^ T cells in tumor sites. BI 2536-induced pyroptosis might have great potential for improving the outcomes of immunotherapy.

## Data availability statement

The original contributions presented in the study are included in the article/**Supplementary Material**. Further ;inquiries can be directed to the corresponding authors.

## Ethics statement

The animal study was reviewed and approved by the Biomedical Ethics Committee of Ruijin Hospital.

## Author contributions

Conceptualization: YZ and LS. Methodology: YZ. Experiment: JH. Data analysis: YS. Visualization: YZ. Supervision: YZ and LS. Writing – original draft: JH, YS, and YZ. Writing – review & editing: YS and LS. All authors contributed to the article and approved the submitted version.

## Acknowledgments

We would like to acknowledge the Institute of Digestive Surgery in Ruijin Hospital for providing the experimental site.

## Conflict of interest

The authors declare that the research was conducted in the absence of any commercial or financial relationships that could be construed as a potential conflict of interest.

## Publisher’s note

All claims expressed in this article are solely those of the authors and do not necessarily represent those of their affiliated organizations, or those of the publisher, the editors and the reviewers. Any product that may be evaluated in this article, or claim that may be made by its manufacturer, is not guaranteed or endorsed by the publisher.
