# Supplementary material for: BI 2536 induces gasdermin E-dependent pyroptosis in ovarian cancer
Source: Front Oncol. 2022 Aug 9;12:963928. doi: 10.3389/fonc.2022.963928 (PMC9396031; doi:10.3389/fonc.2022.963928)
Supplement: Supplementary file 1 [file DataSheet_1.pdf]

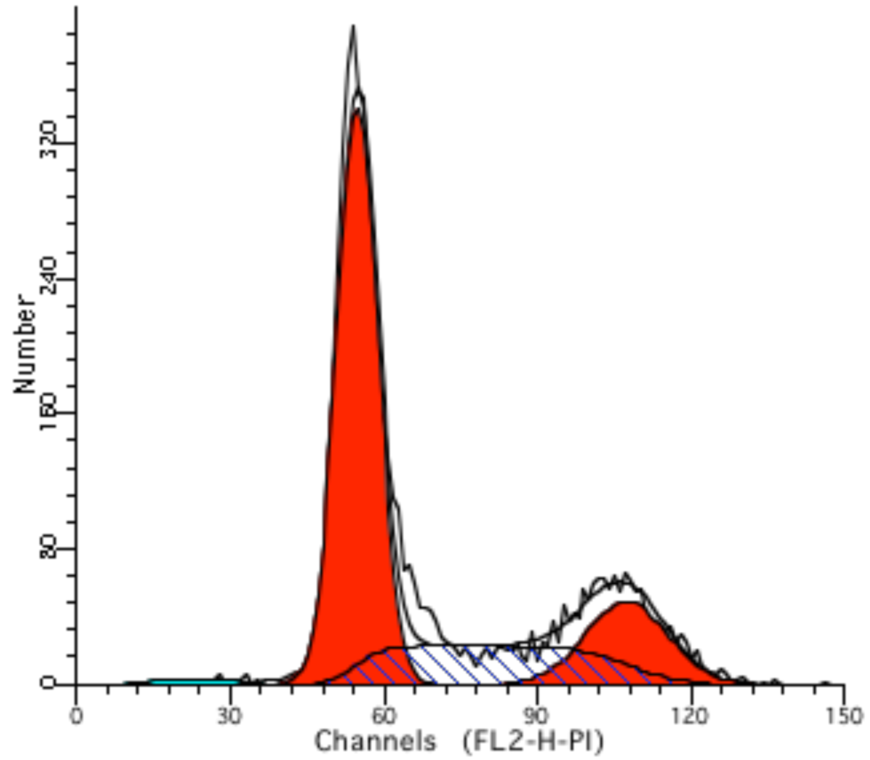

SAMPLE ID: B1-0

File analyzed: 111
Date analyzed: 30-Dec-2021
Model: 1nn0A_DSF
Analysis type: Manual analysis

Diploid: 100.00 %
Dip G1: 61.37 % at 54.79
Dip G2: 17.39 % at 107.43
Dip S: 21.24 % G2/G1: 1.96
%CV: 7.29

Total S-Phase: 21.24 %
Total B.A.D.: 0.00 % no debris no aggs

Apoptosis: 0.93 % Mean: 22.88

Debris: %
Aggregates: 0.00 %
Modeled events: 5643
All cycle events: 5590
Cycle events per channel: 104
RCS: 2.112

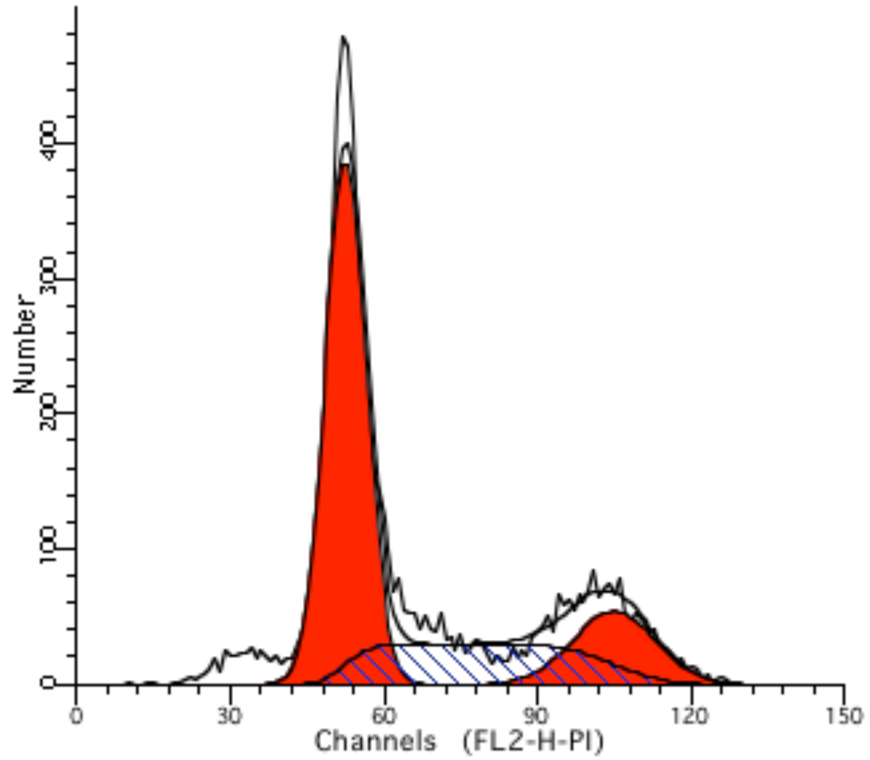

SAMPLE ID: B1-1

File analyzed: 112
Date analyzed: 30-Dec-2021
Model: 1nn0A_DSF
Analysis type: Manual analysis

Diploid: 100.00 %
Dip G1: 59.88 % at 52.53
Dip G2: 16.52 % at 104.83
Dip S: 23.59 % G2/G1: 2.00
%CV: 7.63

Total S-Phase: 23.59 %
Total B.A.D.: 0.00 % no debris no aggs

Apoptosis: % Mean:

Debris: %
Aggregates: 0.00 %
Modeled events: 6506
All cycle events: 6506
Cycle events per channel: 122
RCS: 4.237

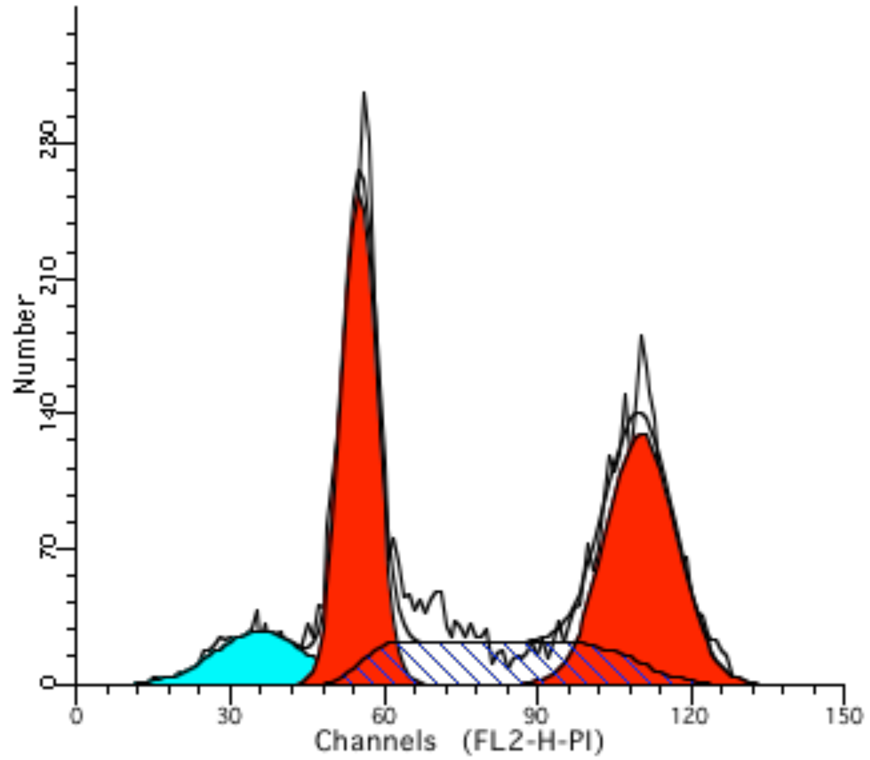

SAMPLE ID: B1-2

File analyzed: 113
Date analyzed: 30-Dec-2021
Model: 1nn0A_DSF
Analysis type: Manual analysis

Diploid: 100.00 %
Dip G1: 38.96 % at 55.16
Dip G2: 39.68 % at 110.16
Dip S: 21.36 % G2/G1: 2.00
%CV: 6.17

Total S-Phase: 21.36 %
Total B.A.D.: 0.00 % no debris no aggs

Apoptosis: 9.64 % Mean: 35.72

Debris: %
Aggregates: 0.00 %
Modeled events: 6174
All cycle events: 5579
Cycle events per channel: 100
RCS: 2.714

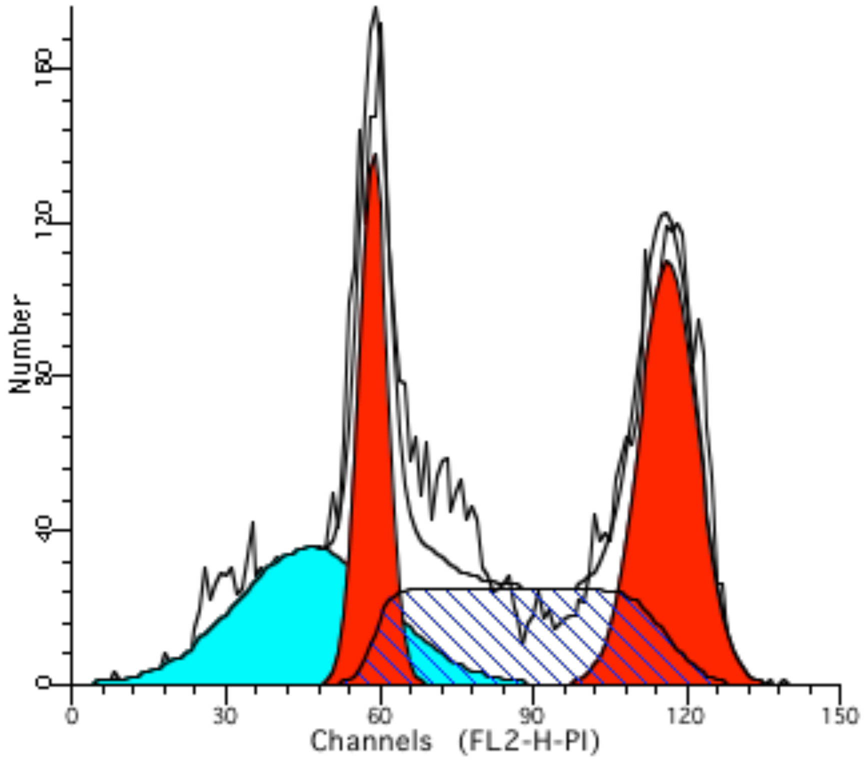

SAMPLE ID: B1-3

File analyzed: 114
Date analyzed: 30-Dec-2021
Model: 1nn0A_DSF
Analysis type: Manual analysis

Diploid: 100.00 %
Dip G1: 24.81 % at 58.73
Dip G2: 39.20 % at 116.27
Dip S: 35.99 % G2/G1: 1.98
%CV: 4.80

Total S-Phase: 35.99 %
Total B.A.D.: 0.00 % no debris no aggs

Apoptosis: 24.55 % Mean: 46.67

Debris: %
Aggregates: 0.00 %
Modeled events: 5258
All cycle events: 3967
Cycle events per channel: 68
RCS: 3.408

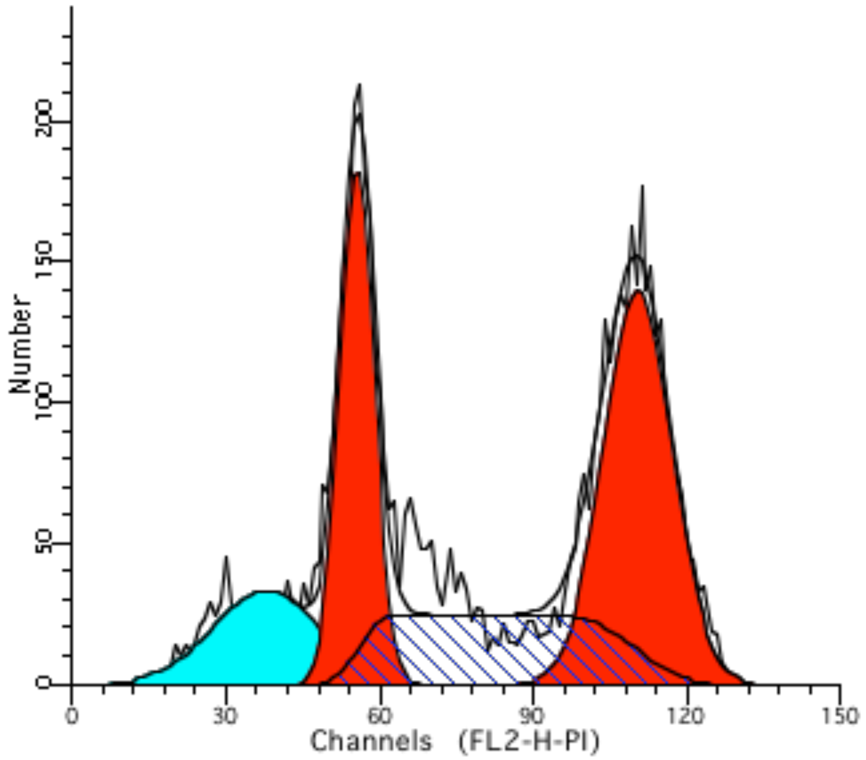

SAMPLE ID: B1-4

File analyzed: 115
Date analyzed: 30-Dec-2021
Model: 1nn0A_DSF
Analysis type: Manual analysis

Diploid: 100.00 %
Dip G1: 29.55 % at 55.57
Dip G2: 45.04 % at 110.35
Dip S: 25.41 %   G2/G1: 1.99
%CV: 6.03

Total S-Phase: 25.41 %
Total B.A.D.: 0.00 %   no debris no aggs

Apoptosis: 14.16 %   Mean: 37.80

Debris: %
Aggregates: 0.00 %
Modeled events: 6083
All cycle events: 5222
Cycle events per channel: 94
RCS: 2.472
