# Supplementary material for: BI 2536 induces gasdermin E-dependent pyroptosis in ovarian cancer
Source: Front Oncol. 2022 Aug 9;12:963928. doi: 10.3389/fonc.2022.963928 (PMC9396031; doi:10.3389/fonc.2022.963928)
Supplement: Supplementary file 2 [file DataSheet_2.pdf]

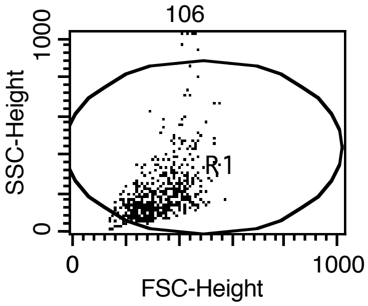

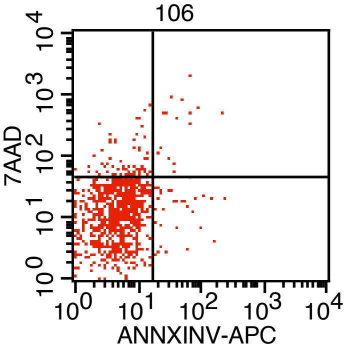

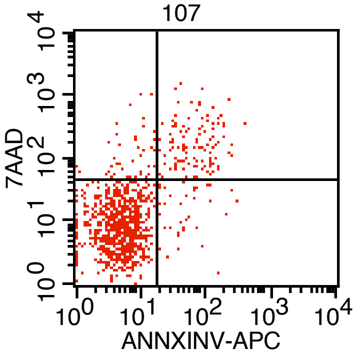

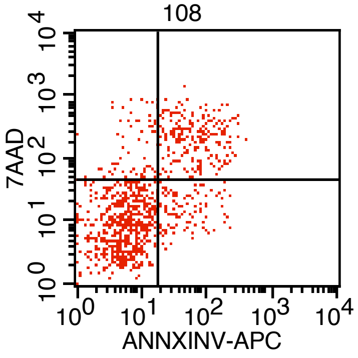

Sample ID: B1-0

Quad	% Gated
UL	7.47
UR	1.98
LL	87.57
LR	2.98

Sample ID: B1-1

Quad	% Gated
UL	4.58
UR	12.52
LL	78.43
LR	4.47

Sample ID: B1-2

Quad	% Gated
UL	7.21
UR	22.79
LL	59.49
LR	10.51

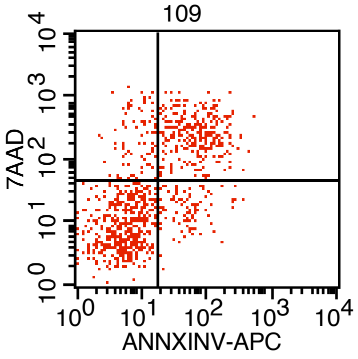

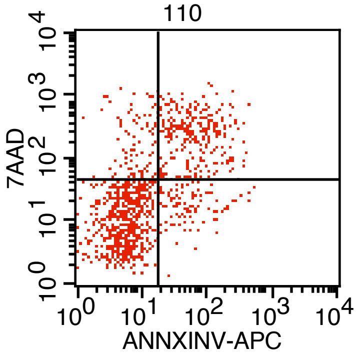

Sample ID: B1-4

Quad	% Gated
UL	8.87
UR	29.51
LL	51.13
LR	10.48

Sample ID: B1-5

Quad	% Gated
UL	9.59
UR	28.64
LL	52.59
LR	9.18
