# Supplementary figures and images for: BI 2536 induces gasdermin E-dependent pyroptosis in ovarian cancer
Source: Front Oncol. 2022 Aug 9;12:963928. doi: 10.3389/fonc.2022.963928 (PMC9396031; doi:10.3389/fonc.2022.963928)

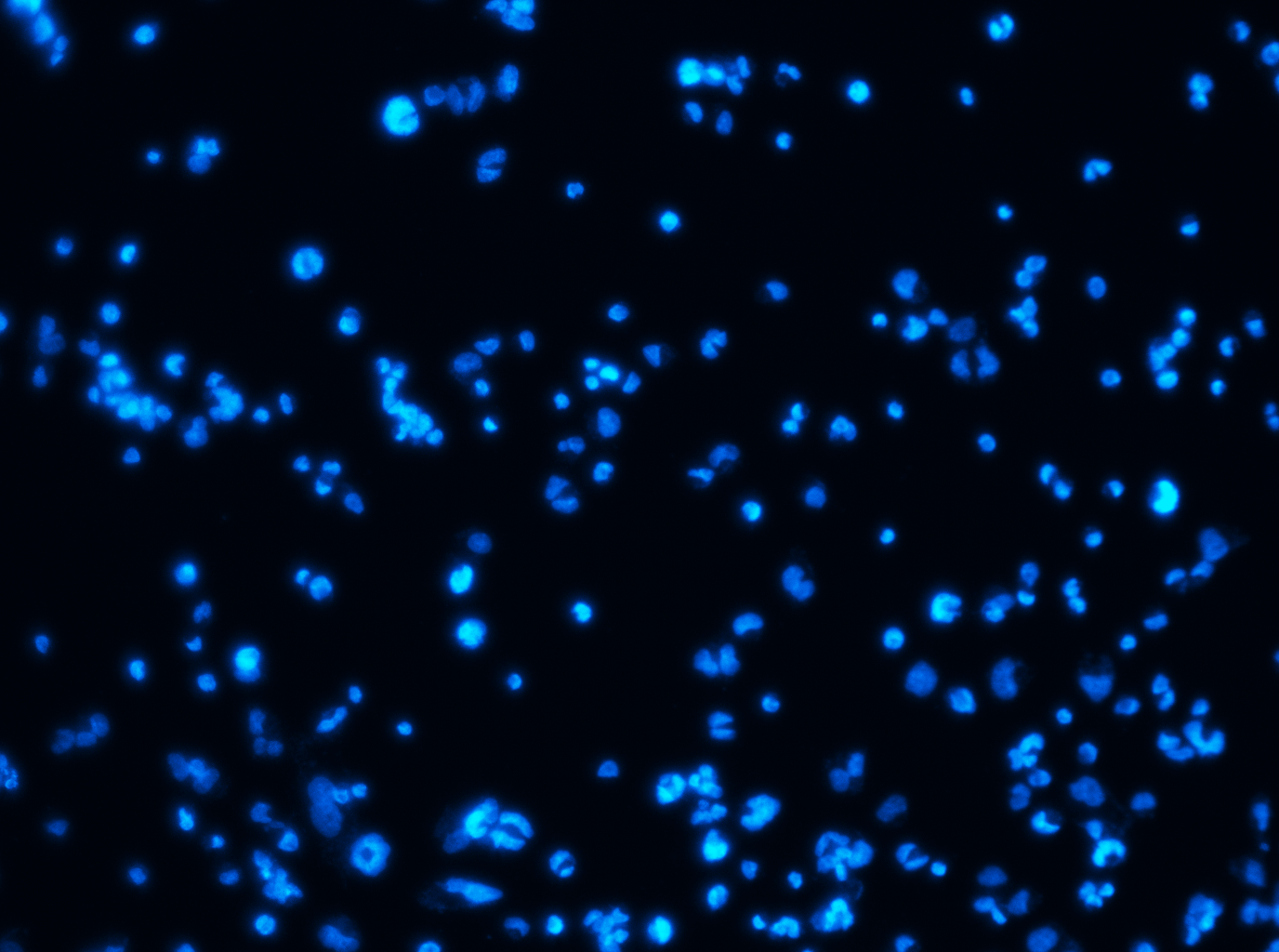

Supplement: Supplementary file 3 [file DataSheet_3.zip › 1E A2780+BI-20X-DAPI.tif]

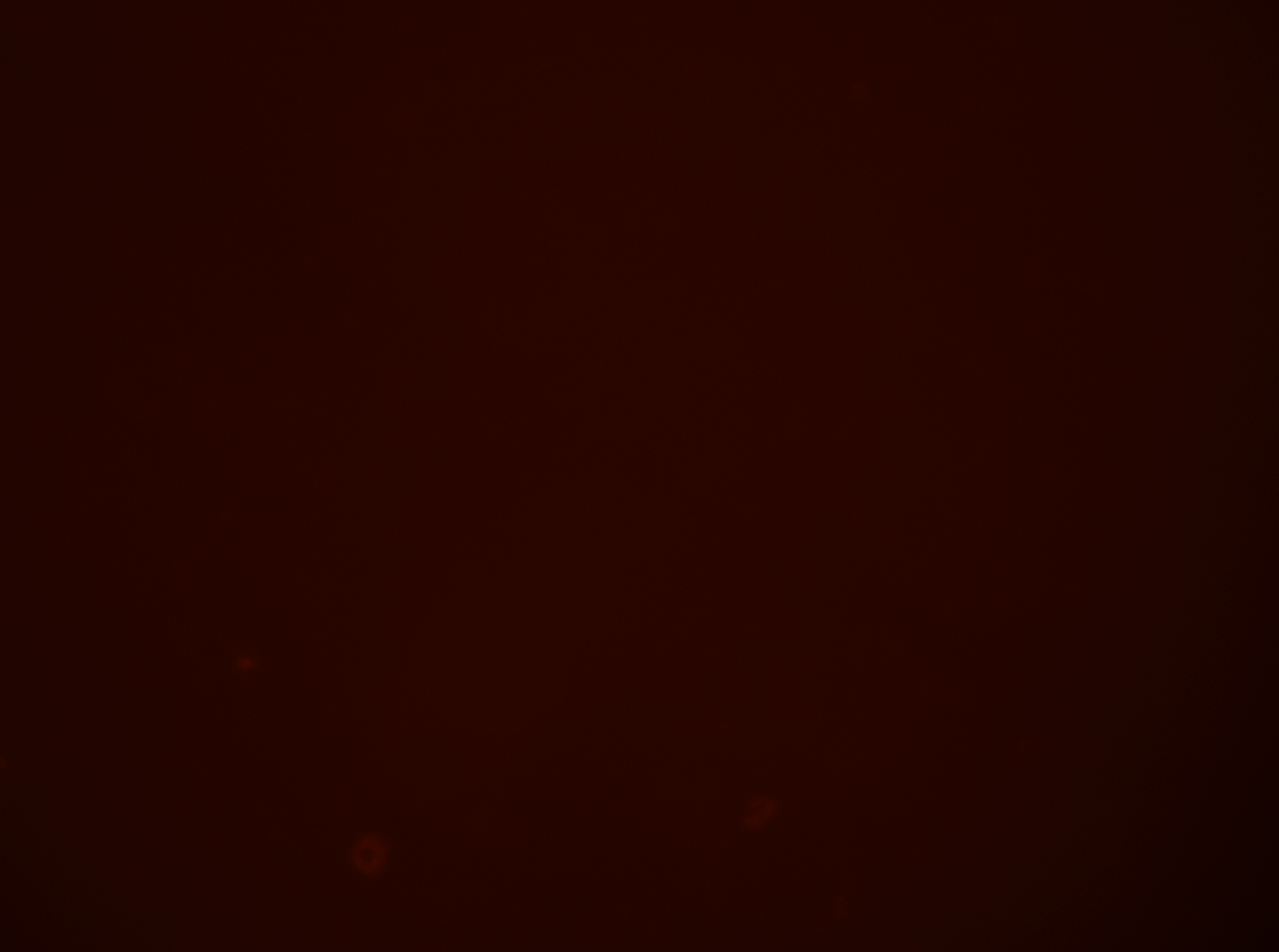

Supplement: Supplementary file 3 [file DataSheet_3.zip › 1E A2780+BI-20X-EDU.tif]

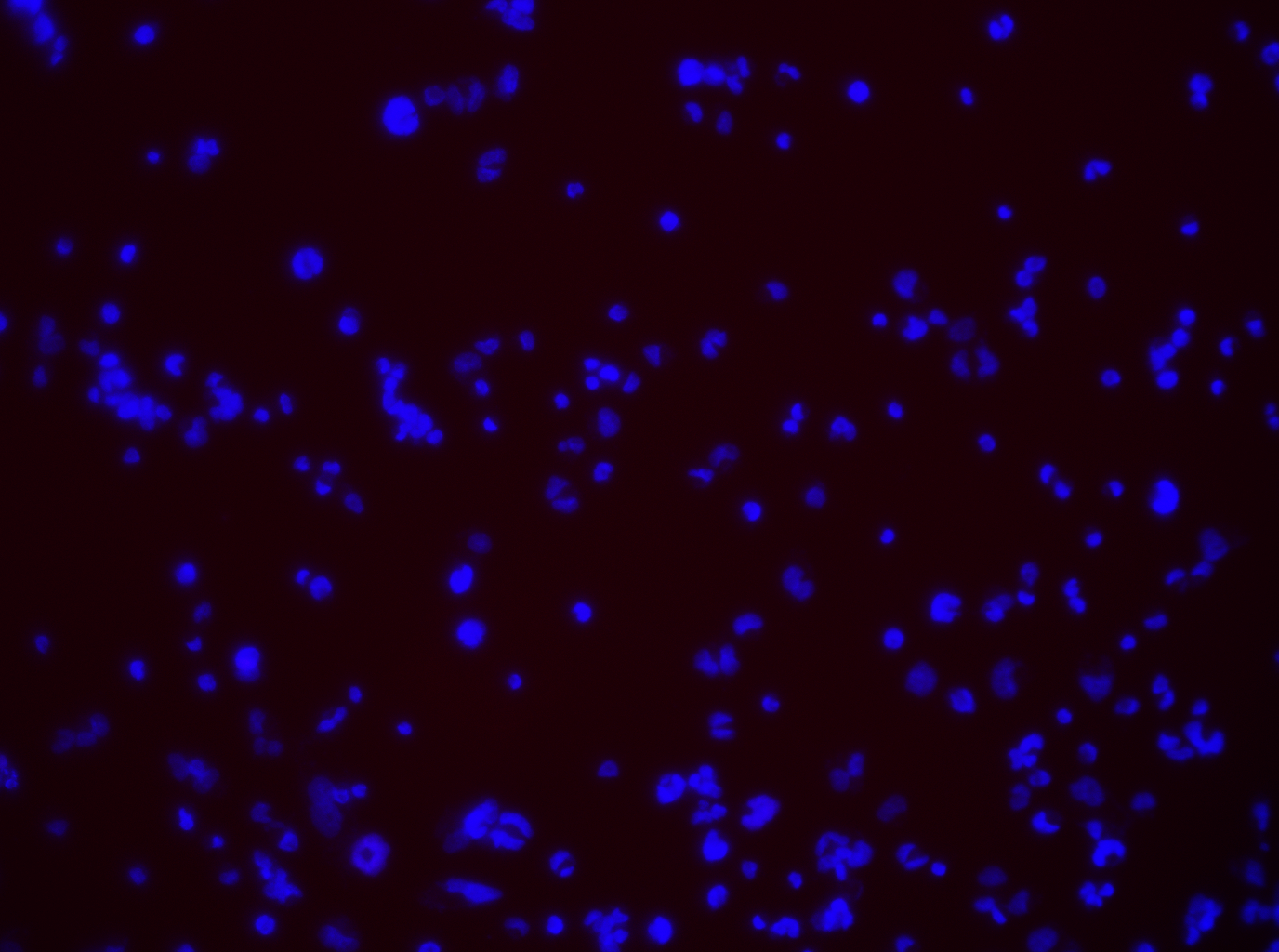

Supplement: Supplementary file 3 [file DataSheet_3.zip › 1E A2780+BI-20X-EDU-MERGE.tif]

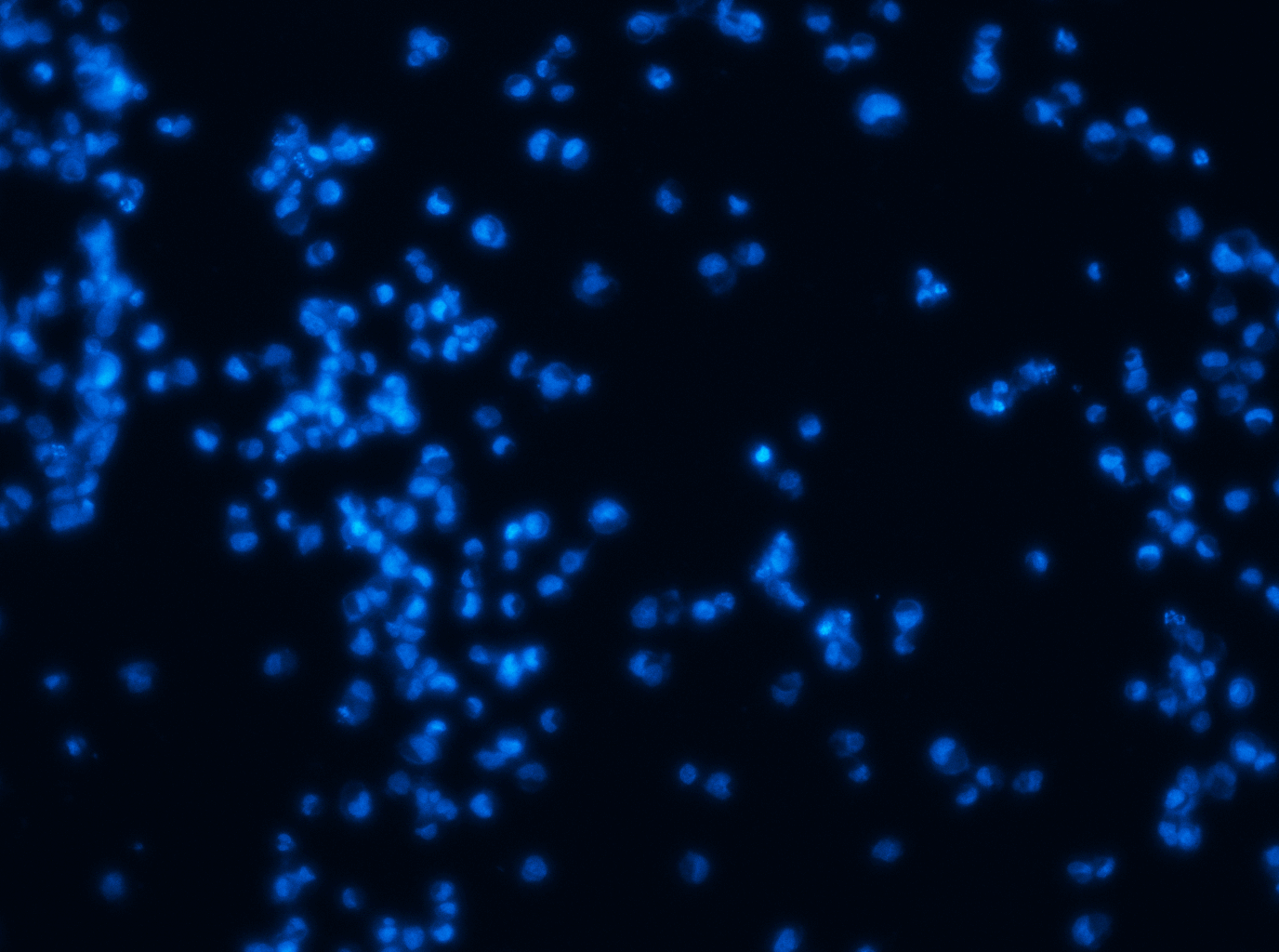

Supplement: Supplementary file 3 [file DataSheet_3.zip › 1E A2780-CTRL-20X-DAPI.tif]

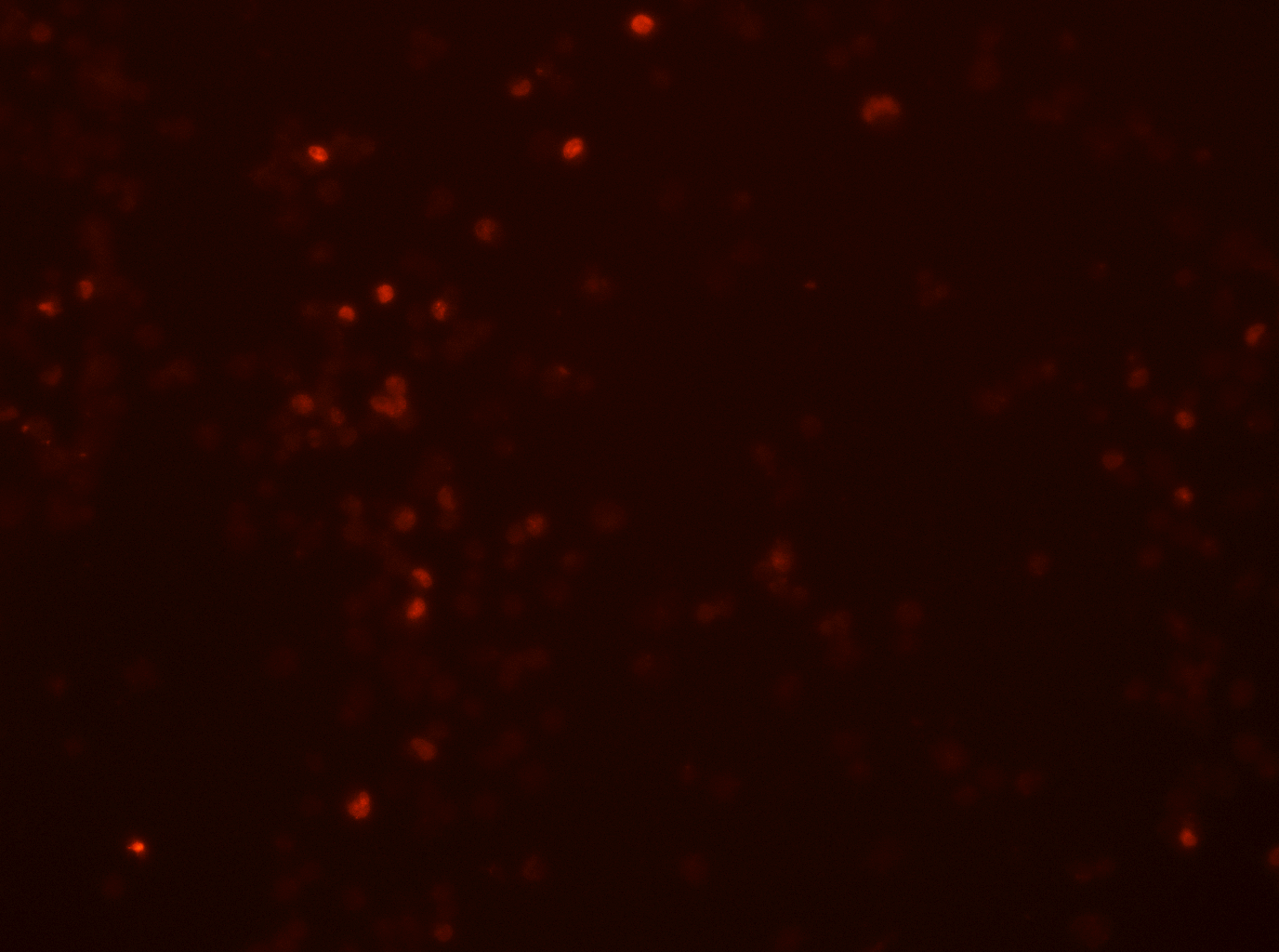

Supplement: Supplementary file 3 [file DataSheet_3.zip › 1E A2780-CTRL-20X-EDU.tif]

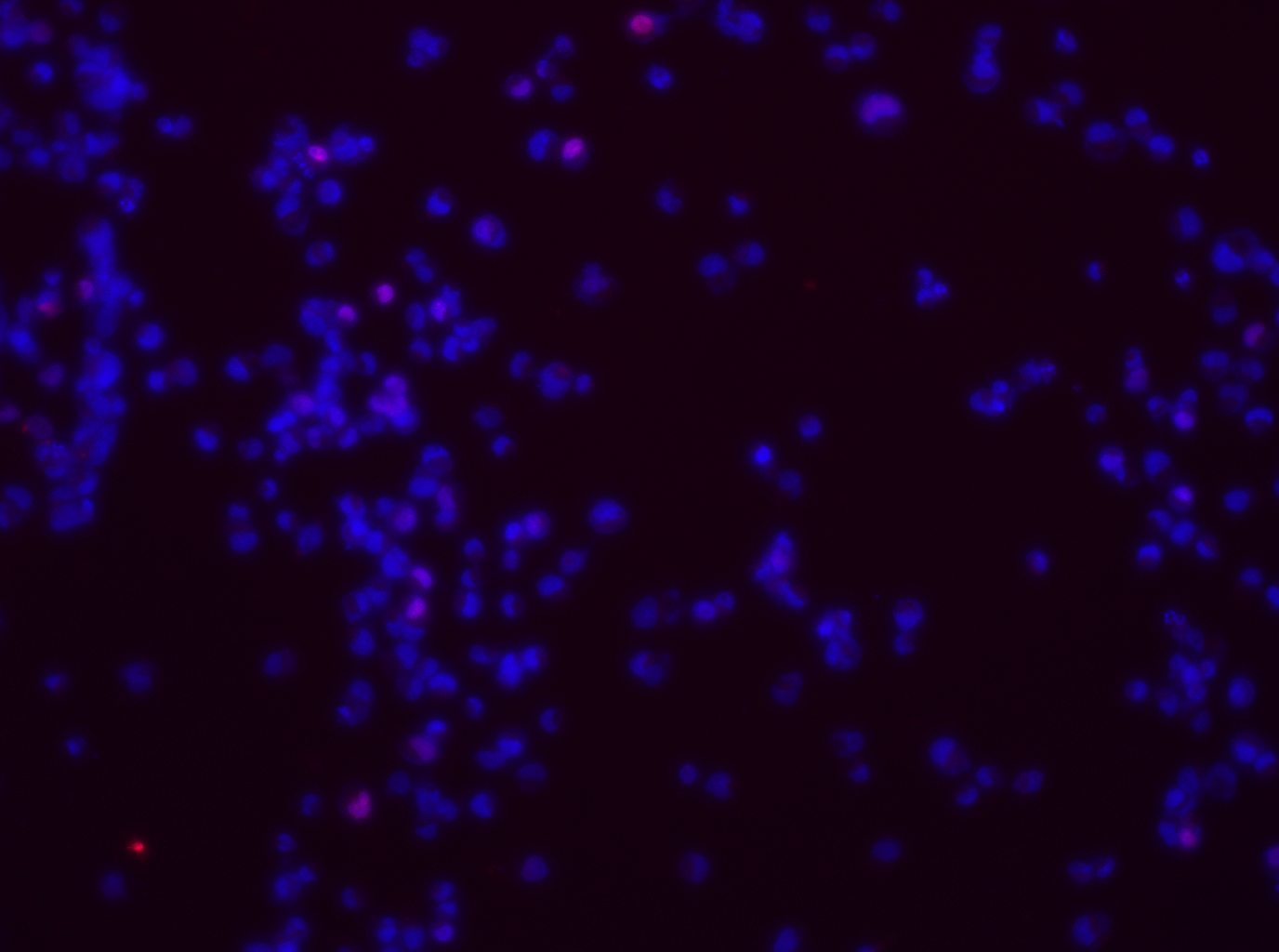

Supplement: Supplementary file 3 [file DataSheet_3.zip › 1E A2780-CTRL-20X-EDU-MERGE.tif]

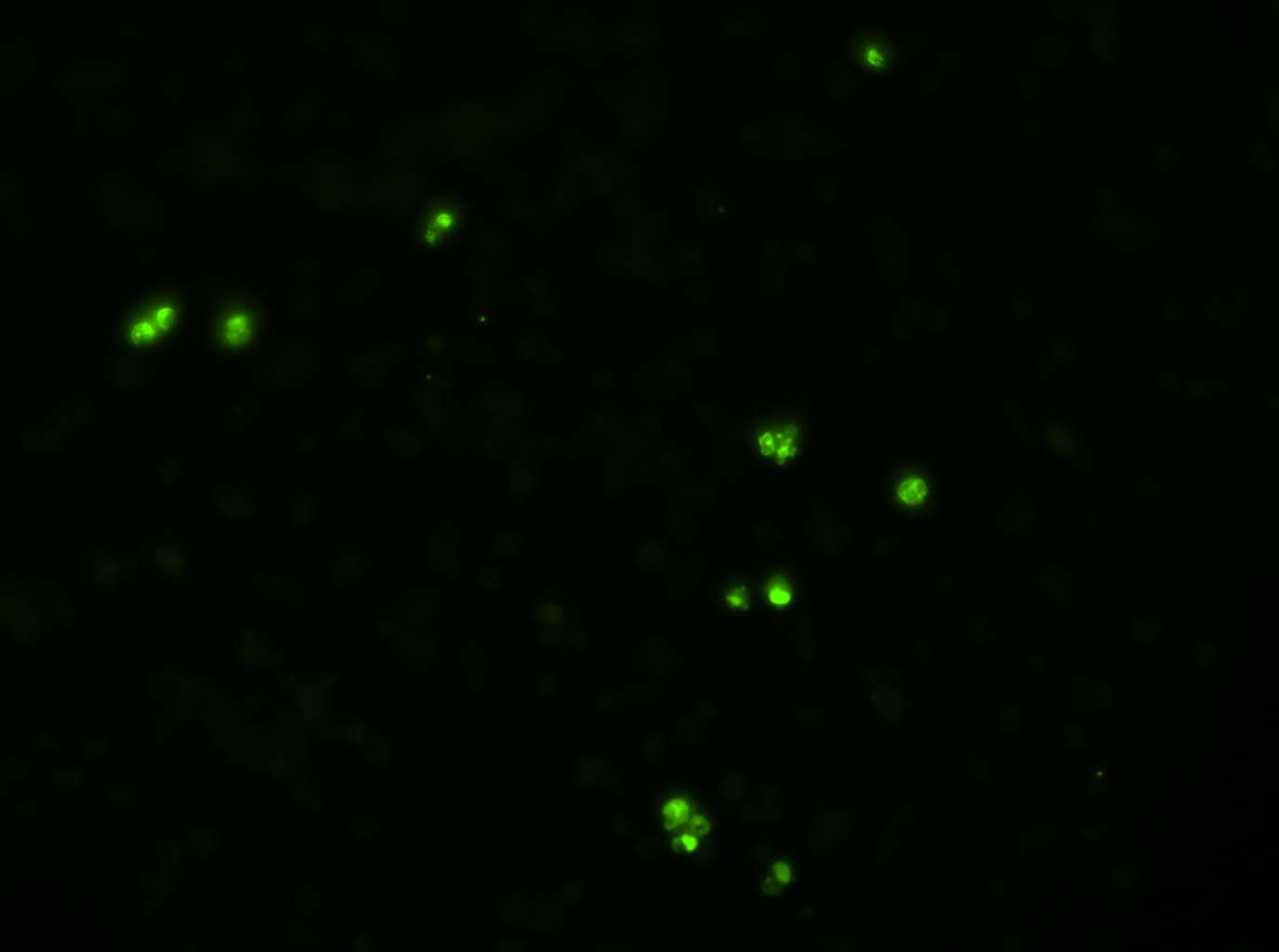

Supplement: Supplementary file 3 [file DataSheet_3.zip › 2C A2780+BI-20X-10_RGB_FITC.tif]

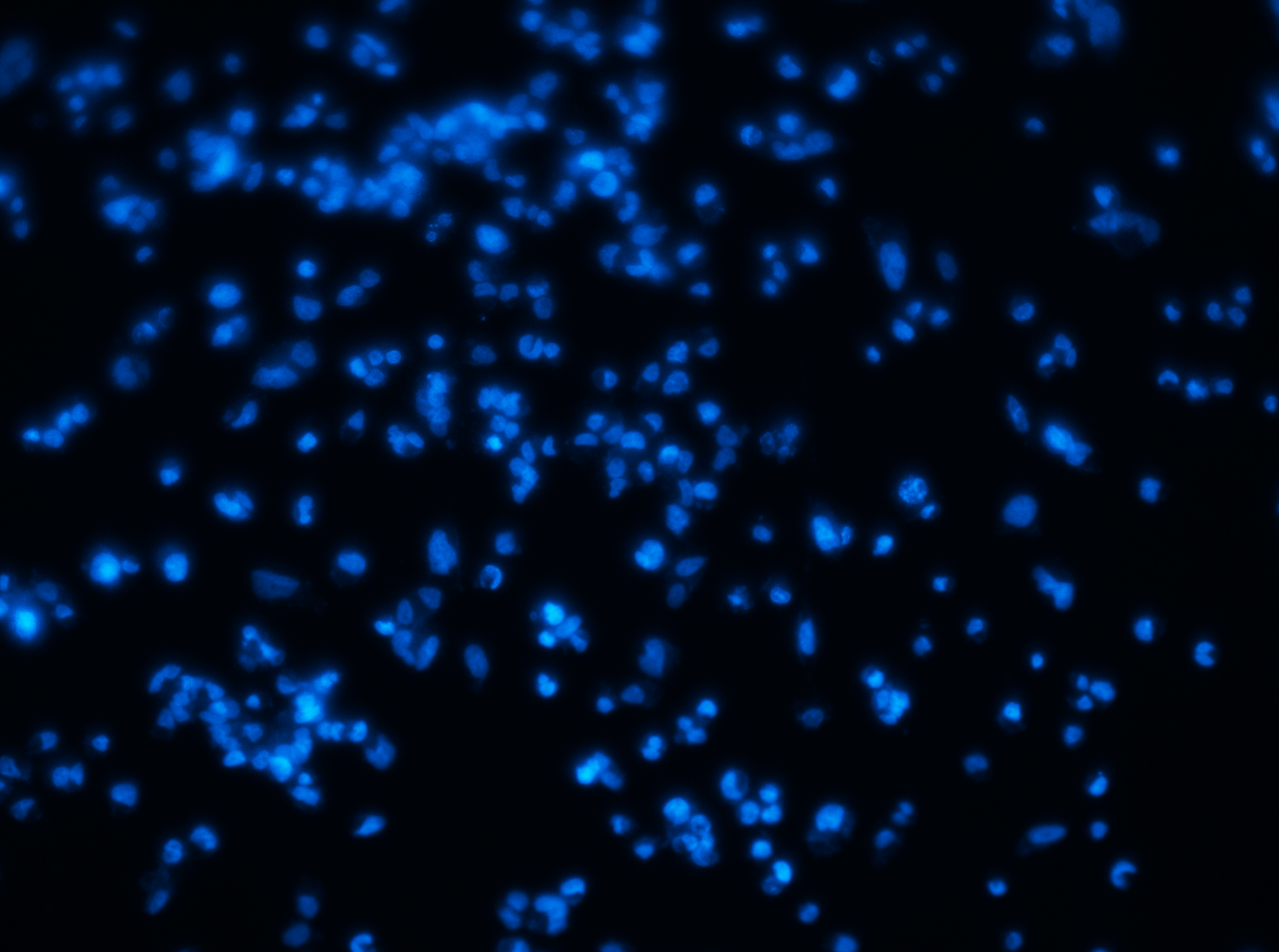

Supplement: Supplementary file 3 [file DataSheet_3.zip › 2C A2780+BI-20X-DAPI.tif]

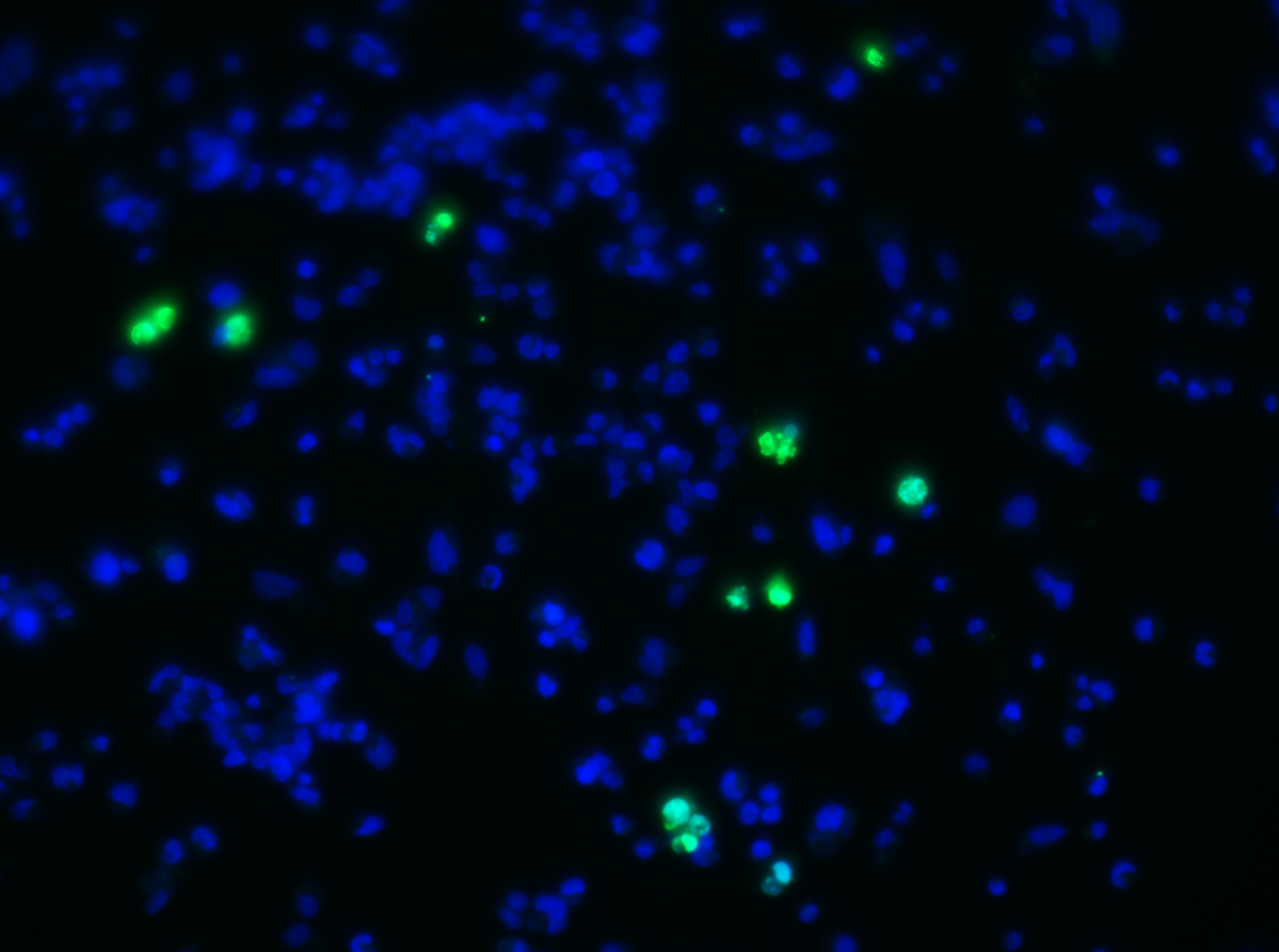

Supplement: Supplementary file 3 [file DataSheet_3.zip › 2C A2780+BI-20X-MERGE.tif]

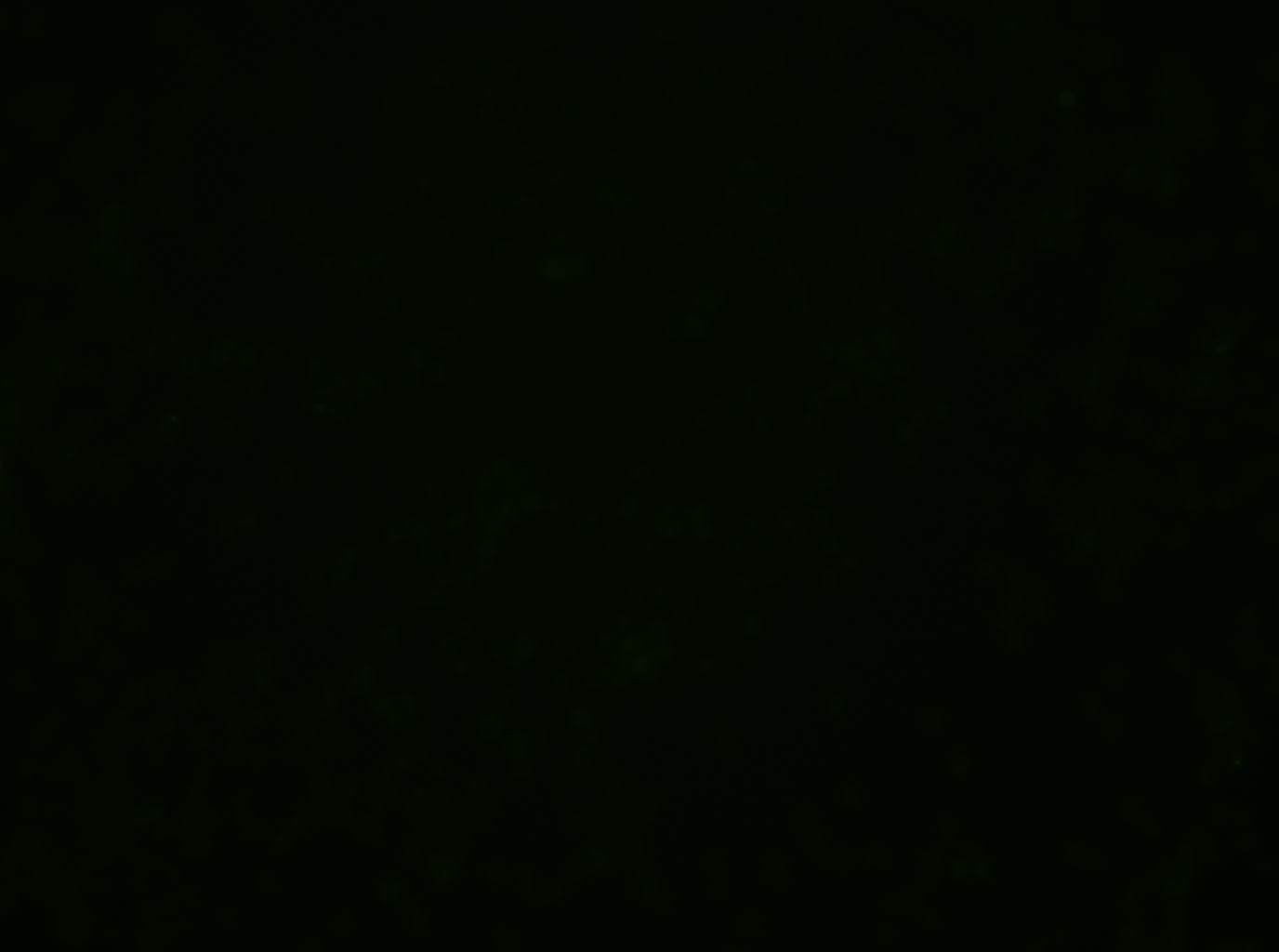

Supplement: Supplementary file 3 [file DataSheet_3.zip › 2C A2780-CTRL-20X-4_RGB_FITC.tif]

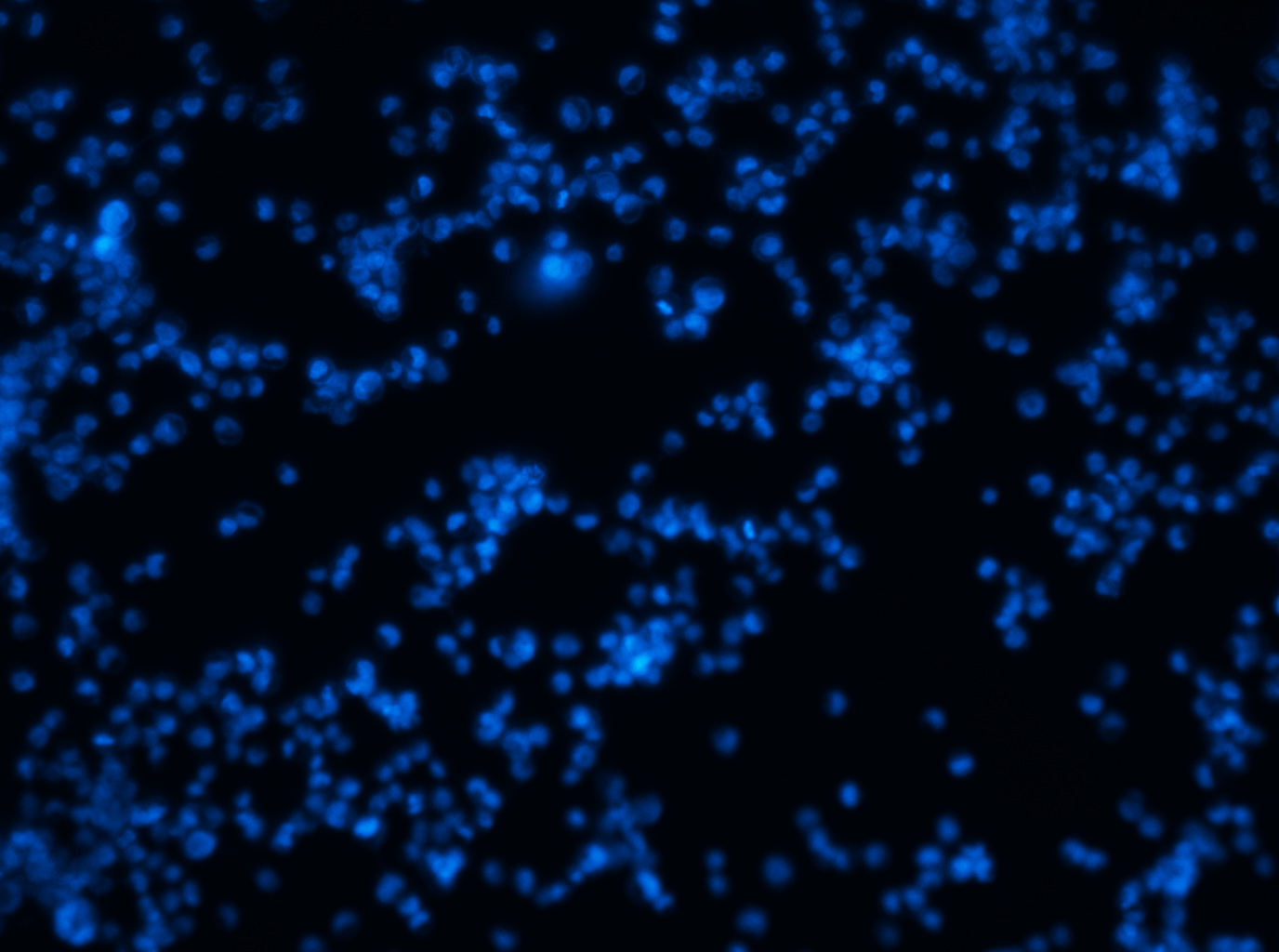

Supplement: Supplementary file 3 [file DataSheet_3.zip › 2C A2780-CTRL-20X-DAPI.tif]

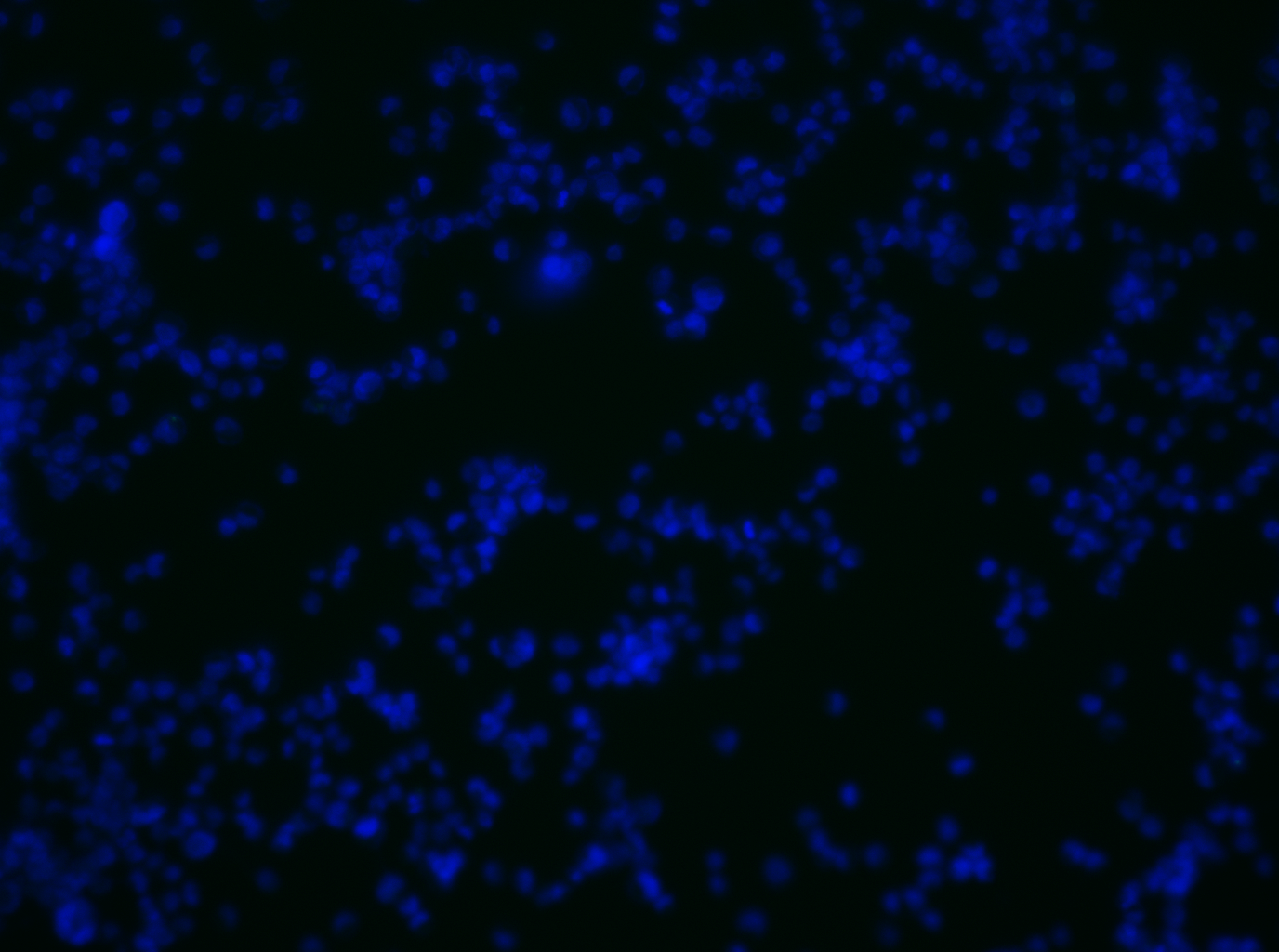

Supplement: Supplementary file 3 [file DataSheet_3.zip › 2C A2780-CTRL-20X-MERGE.tif]

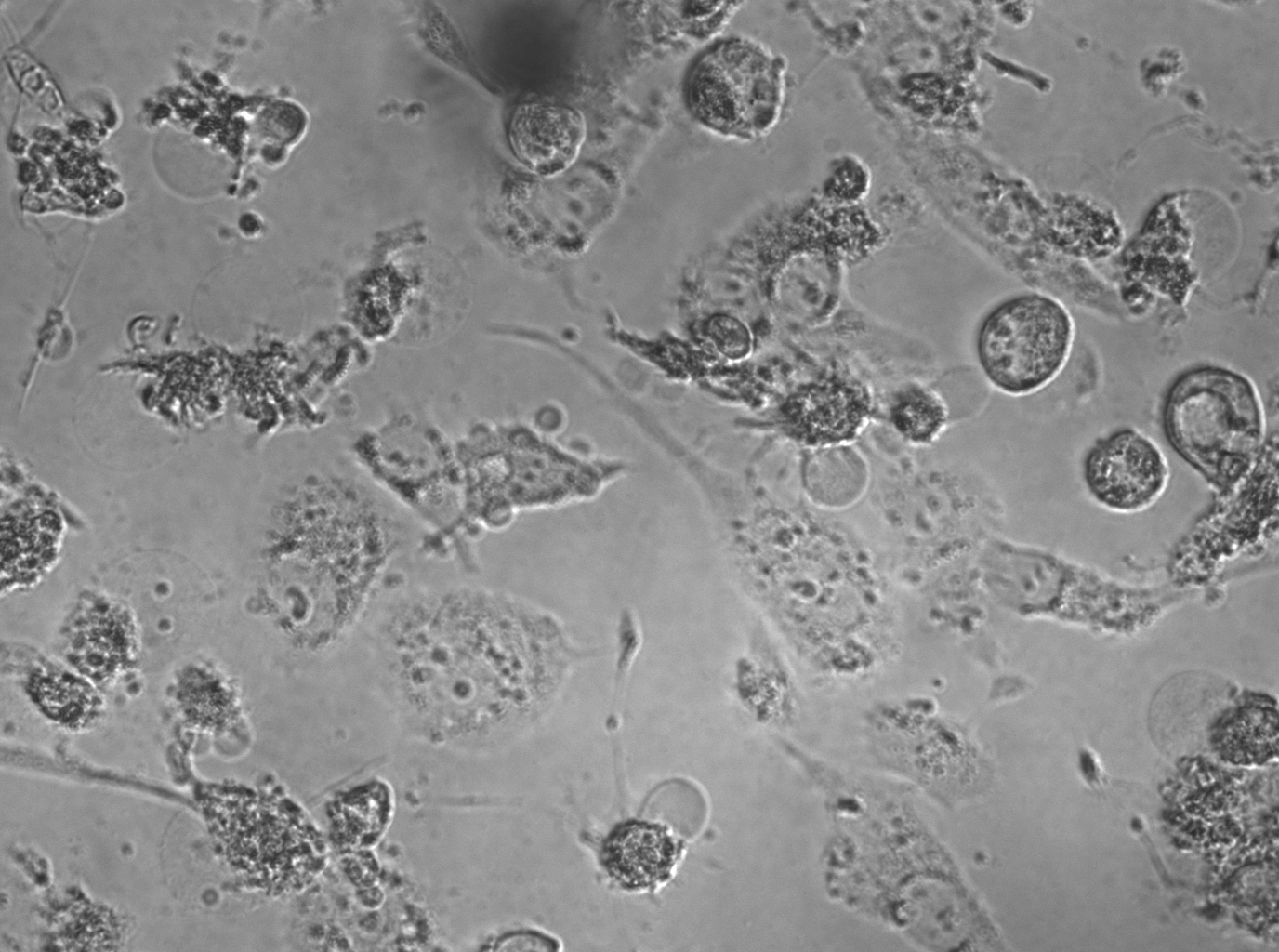

Supplement: Supplementary file 4 [file DataSheet_4.zip › 3A A2780+BI-40X-3_RGB_Empty.tif]

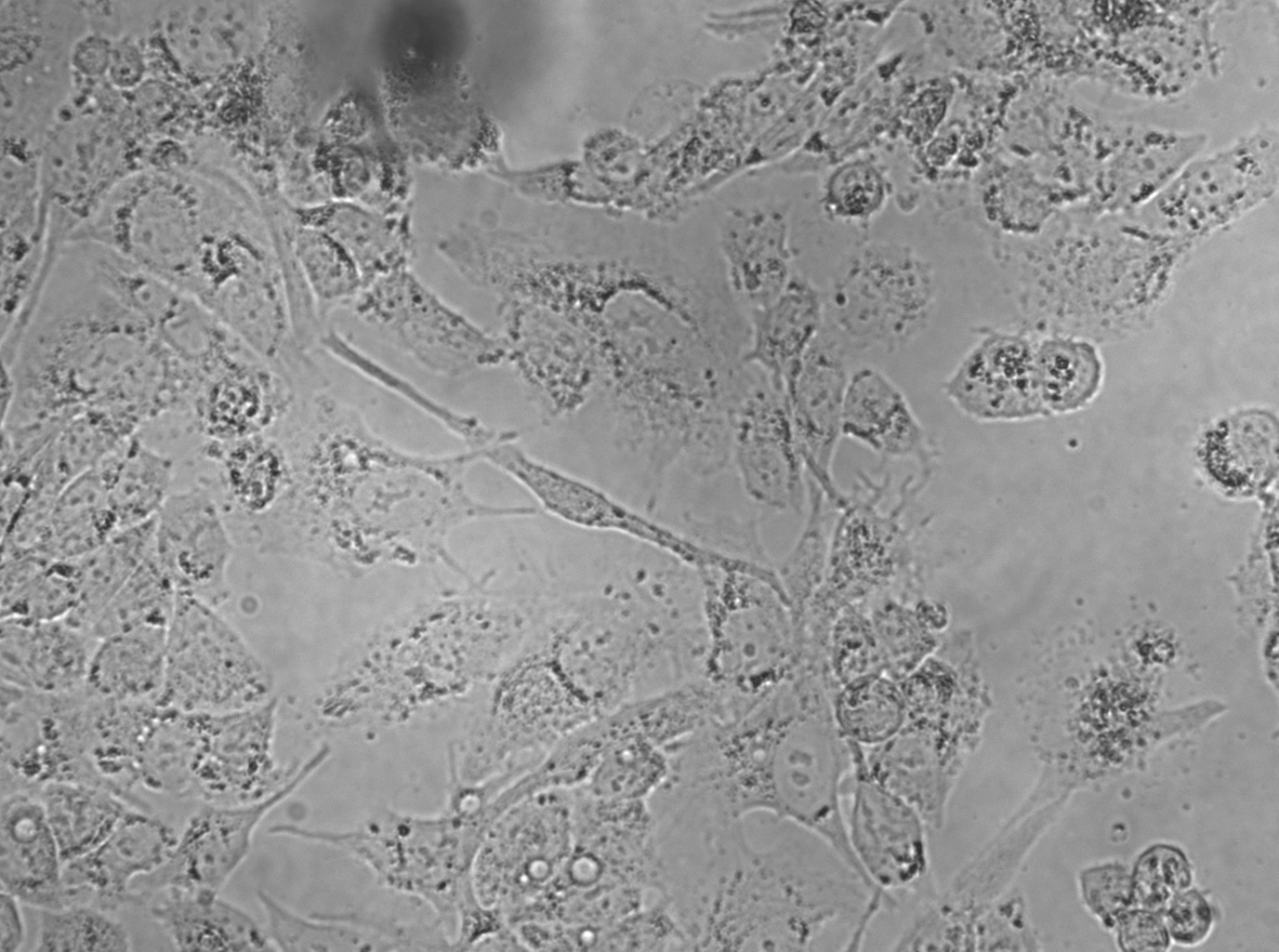

Supplement: Supplementary file 4 [file DataSheet_4.zip › 3A A2780-40X-1_RGB_Empty.tif]

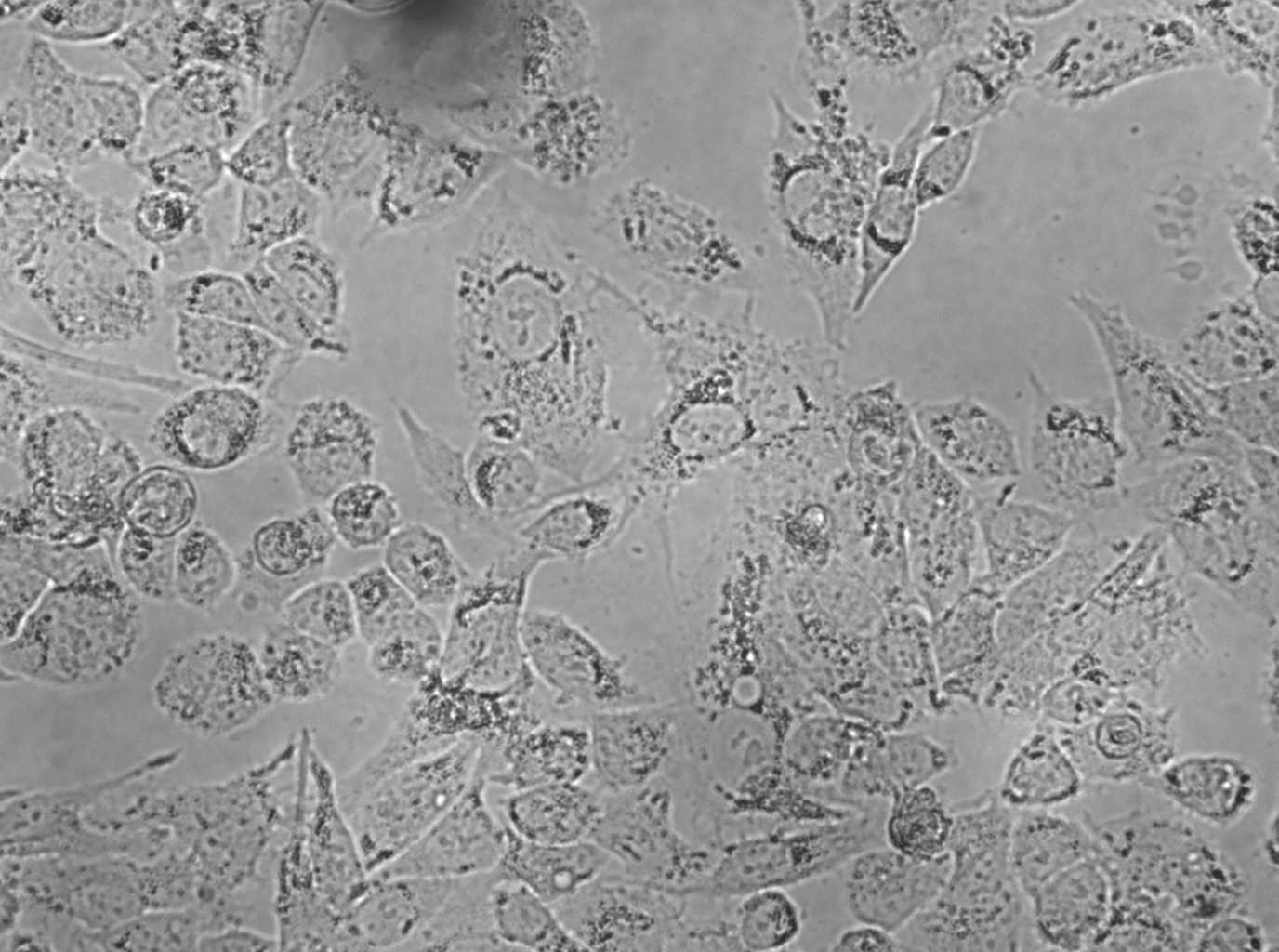

Supplement: Supplementary file 4 [file DataSheet_4.zip › 4A A2780+BI+DEVD-40X-3_RGB_Empty.tif]

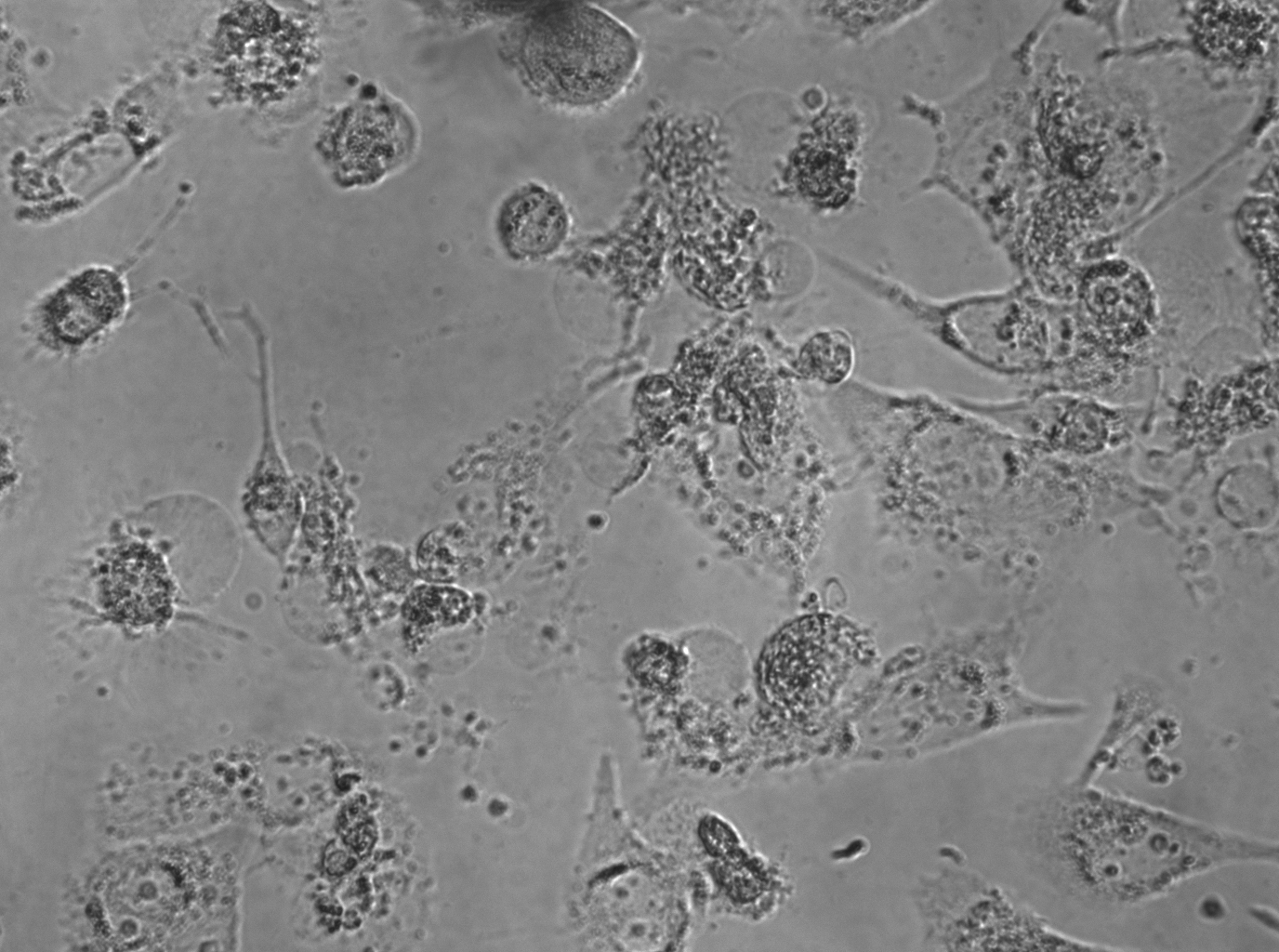

Supplement: Supplementary file 4 [file DataSheet_4.zip › 4A A2780+BI-40X-2_RGB_Empty.tif]

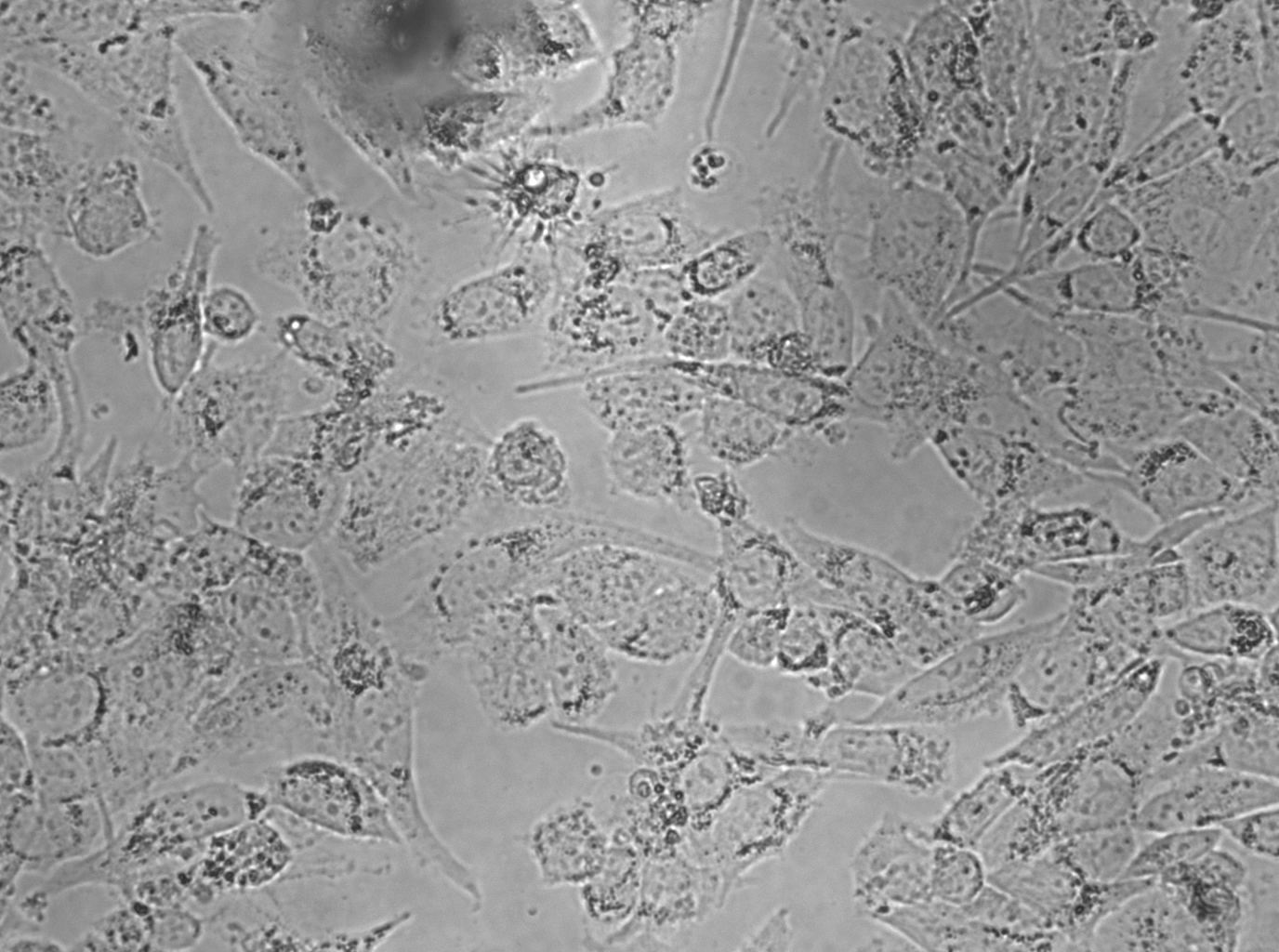

Supplement: Supplementary file 4 [file DataSheet_4.zip › 4A A2780-40X-2_RGB_Empty.tif]

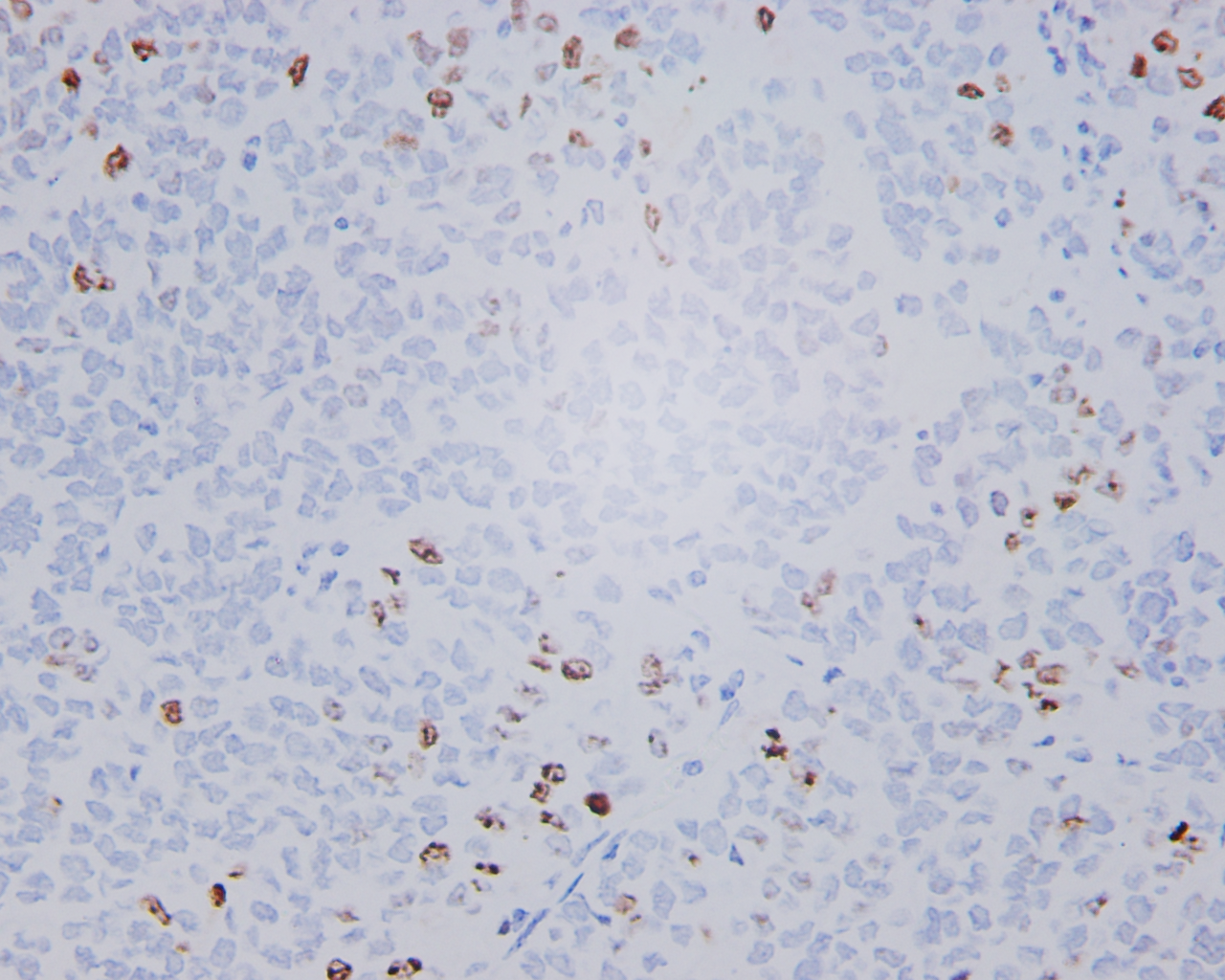

Supplement: Supplementary file 4 [file DataSheet_4.zip › 5C A2780 BI-Ki67-20X-4.tif]

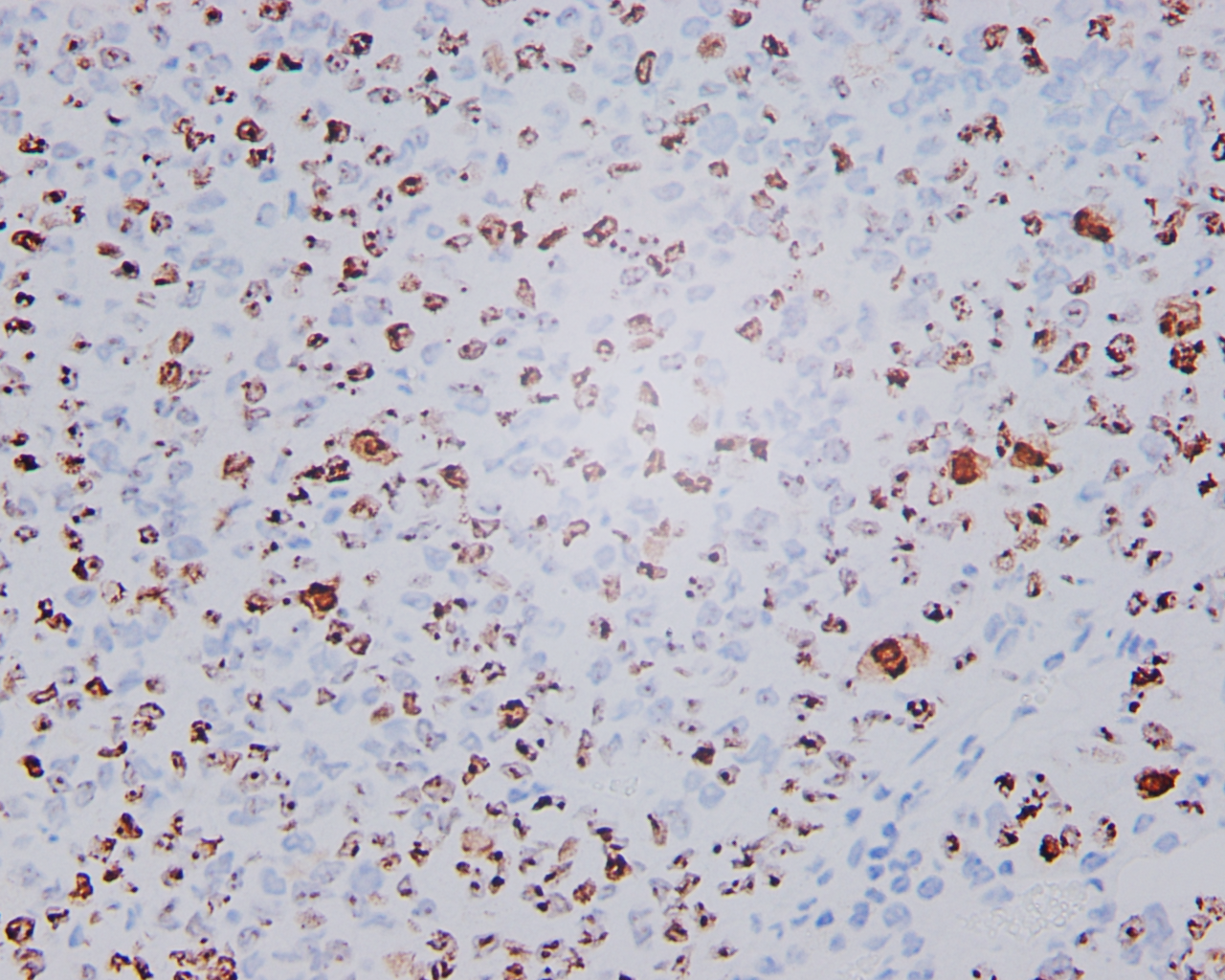

Supplement: Supplementary file 4 [file DataSheet_4.zip › 5C A2780 CTRL-Ki67-20X-3.tif]

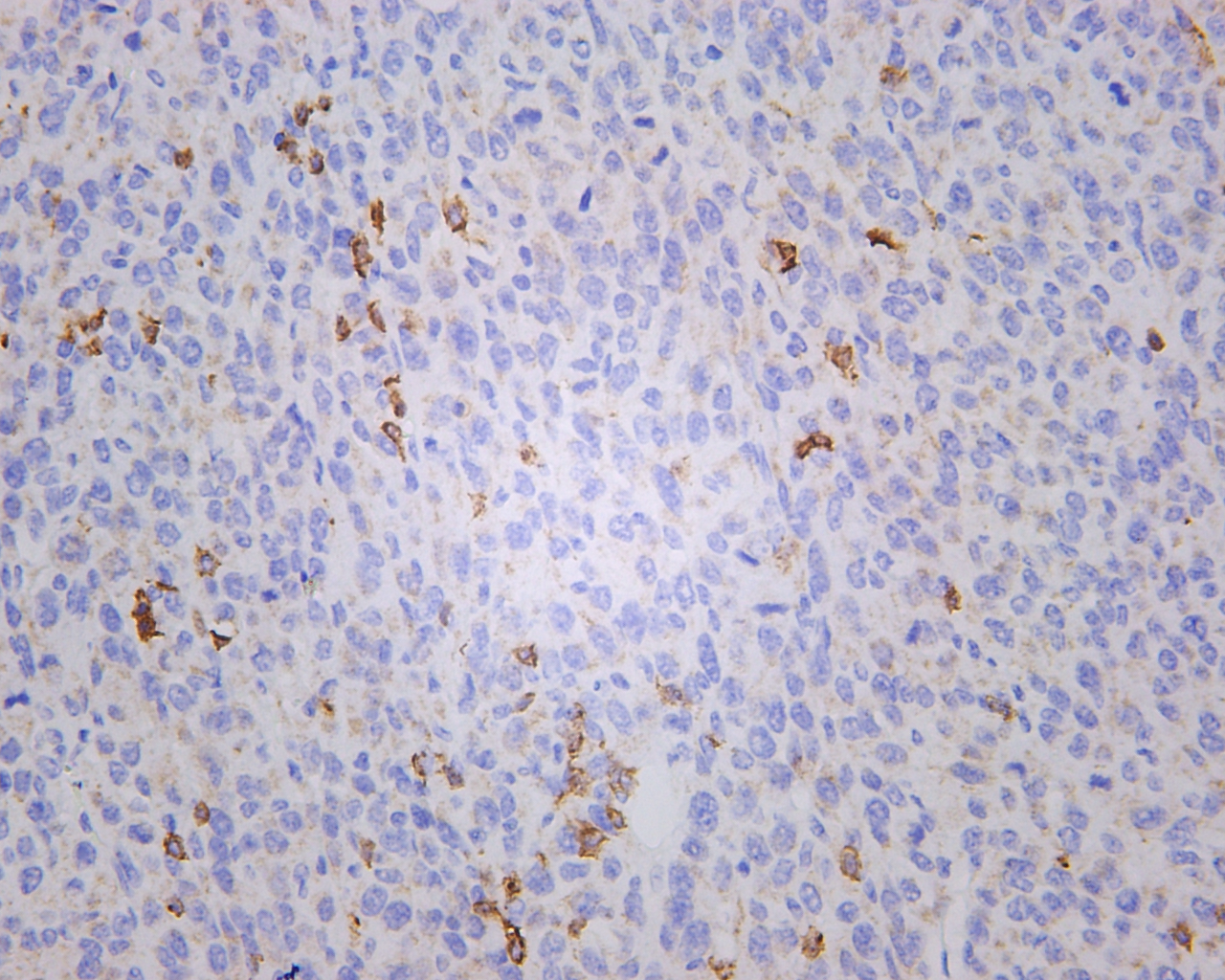

Supplement: Supplementary file 4 [file DataSheet_4.zip › 5C BALBC-BI-CD8-20X.tif]

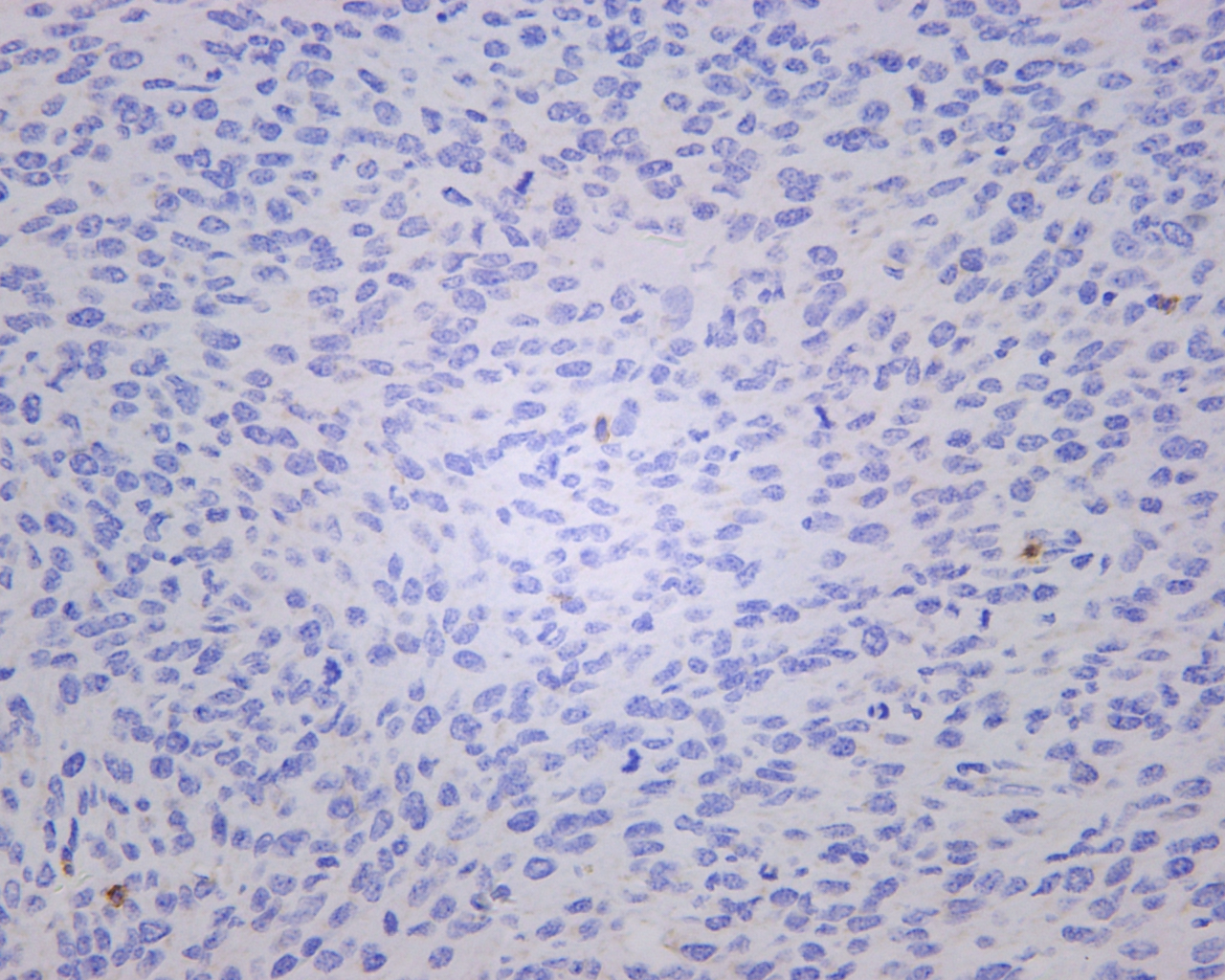

Supplement: Supplementary file 4 [file DataSheet_4.zip › 5C BALBC-CTRL-CD8-20X.tif]

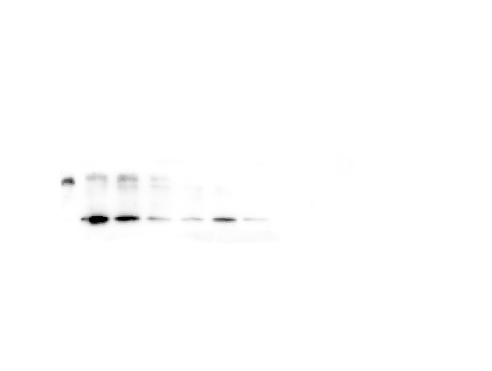

Supplement: Supplementary file 5 [file DataSheet_5.zip › 2D Bcl-2.png]

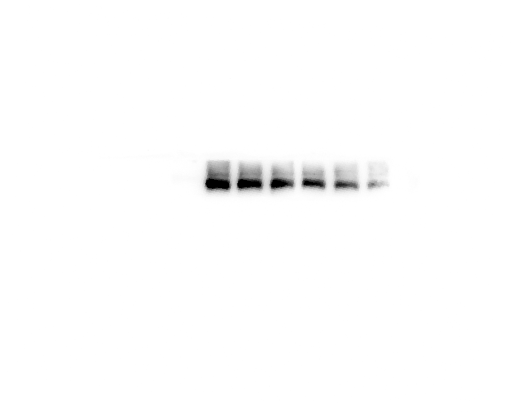

Supplement: Supplementary file 5 [file DataSheet_5.zip › 2D cIAP1.png]

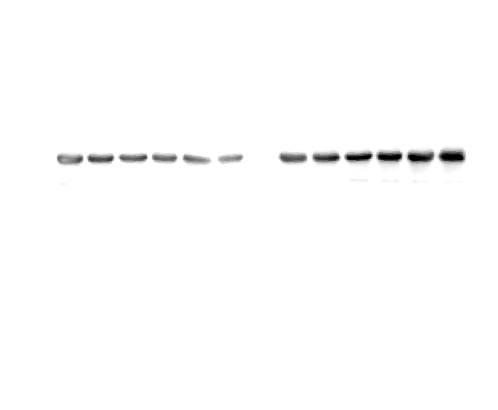

Supplement: Supplementary file 5 [file DataSheet_5.zip › 2D GAPDH-2.png]

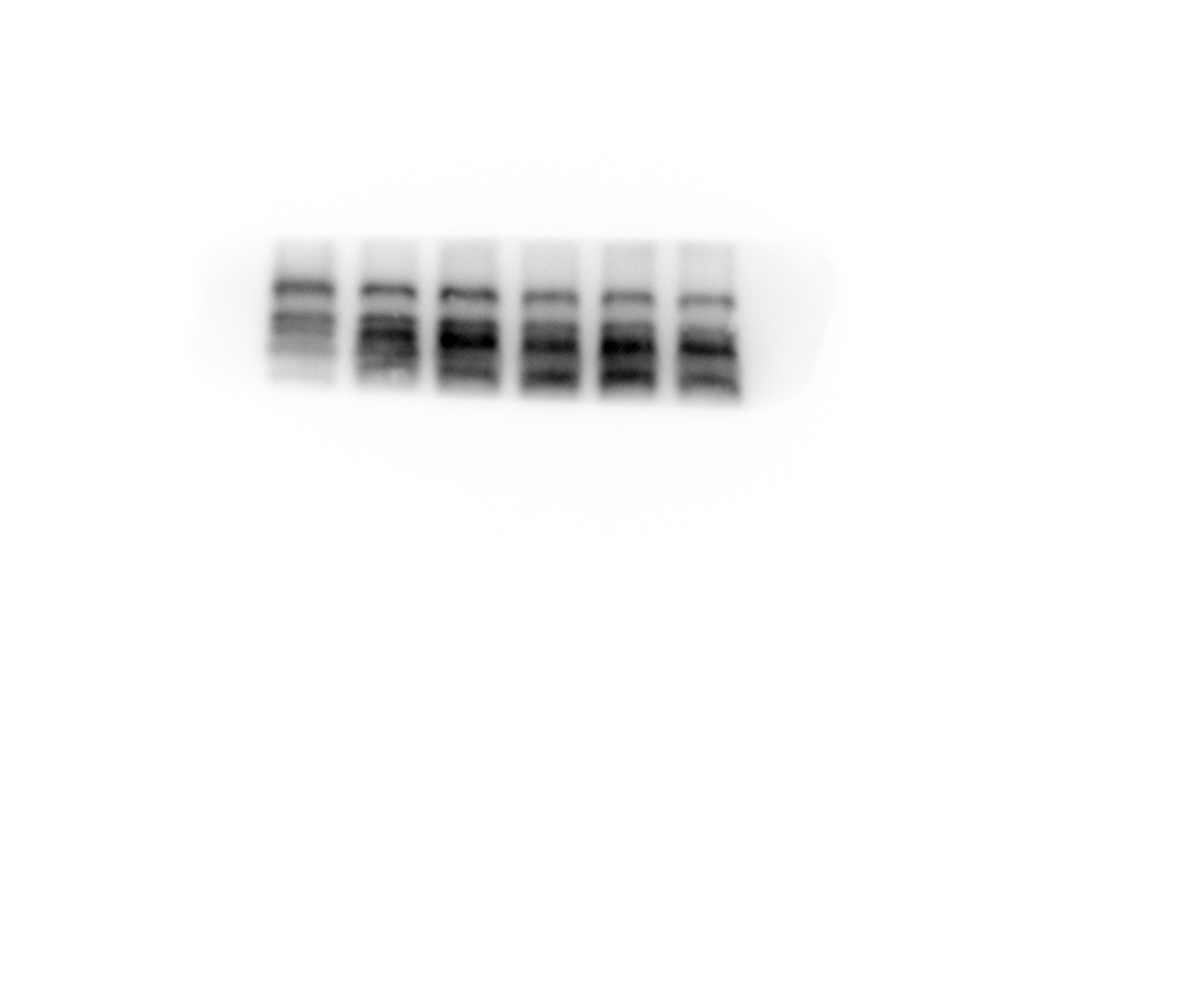

Supplement: Supplementary file 5 [file DataSheet_5.zip › 3D C-CASP3-6.Tif]

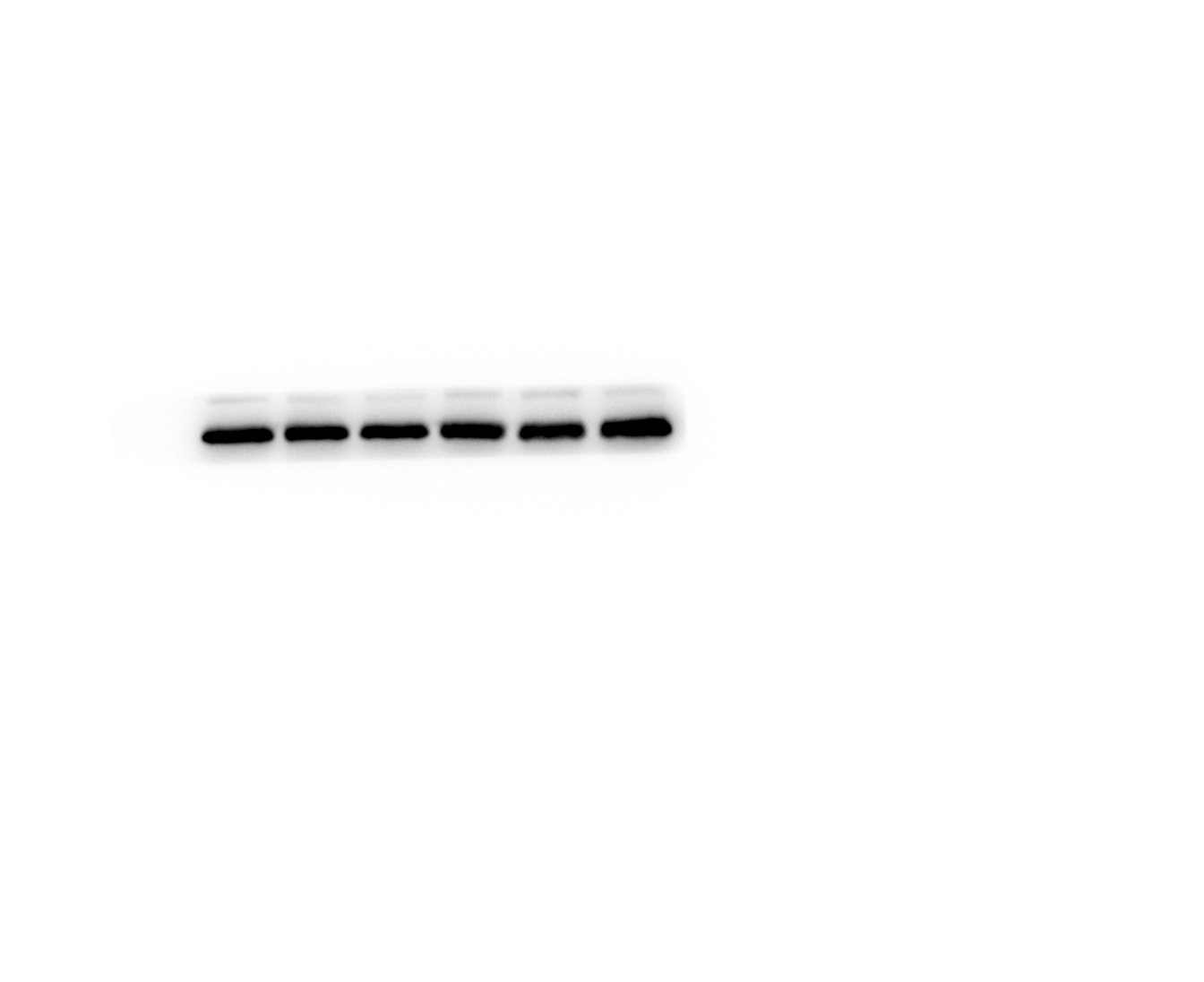

Supplement: Supplementary file 5 [file DataSheet_5.zip › 3D GAPDH-4.Tif]

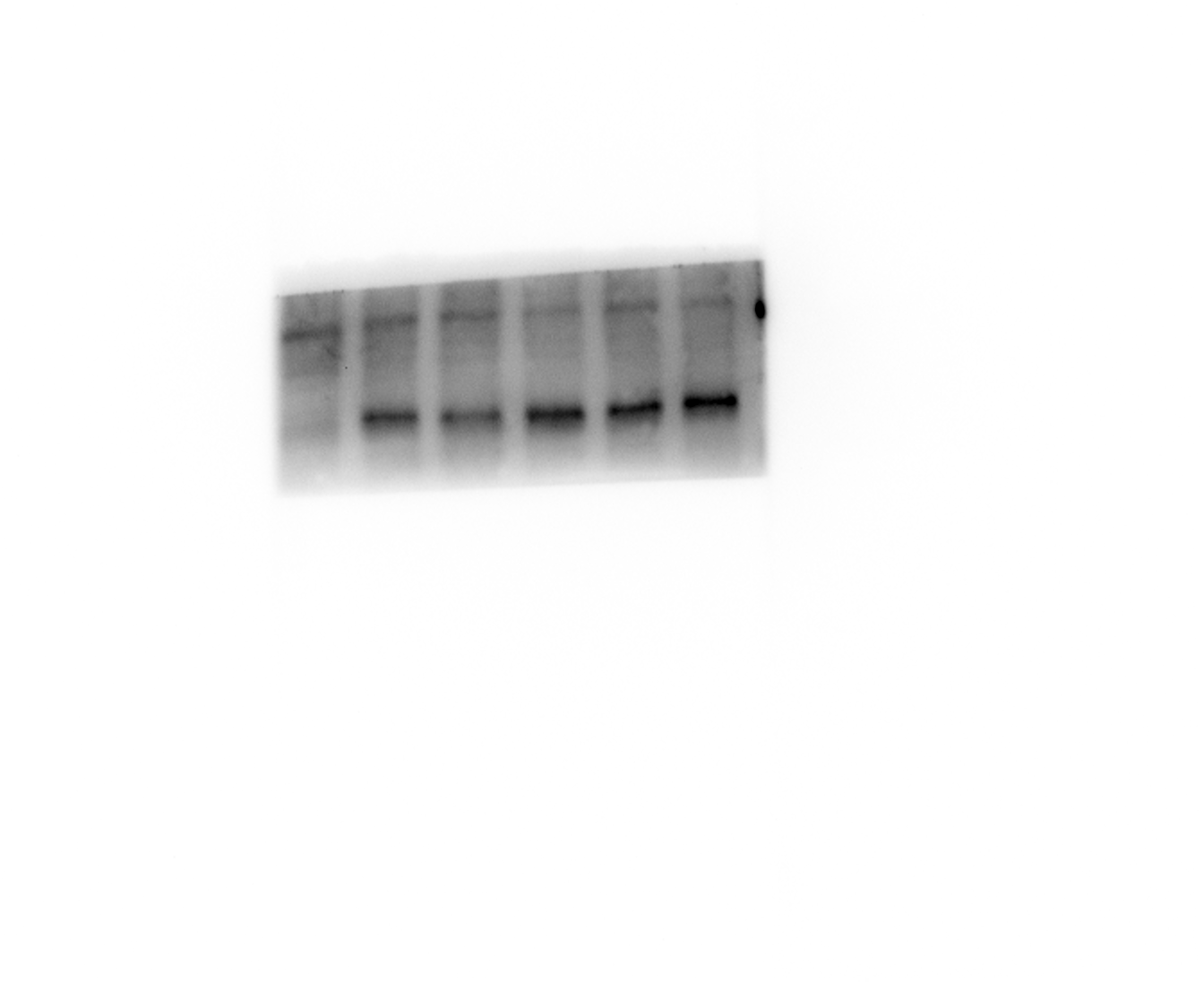

Supplement: Supplementary file 5 [file DataSheet_5.zip › 3D GSDME-4.Tif]

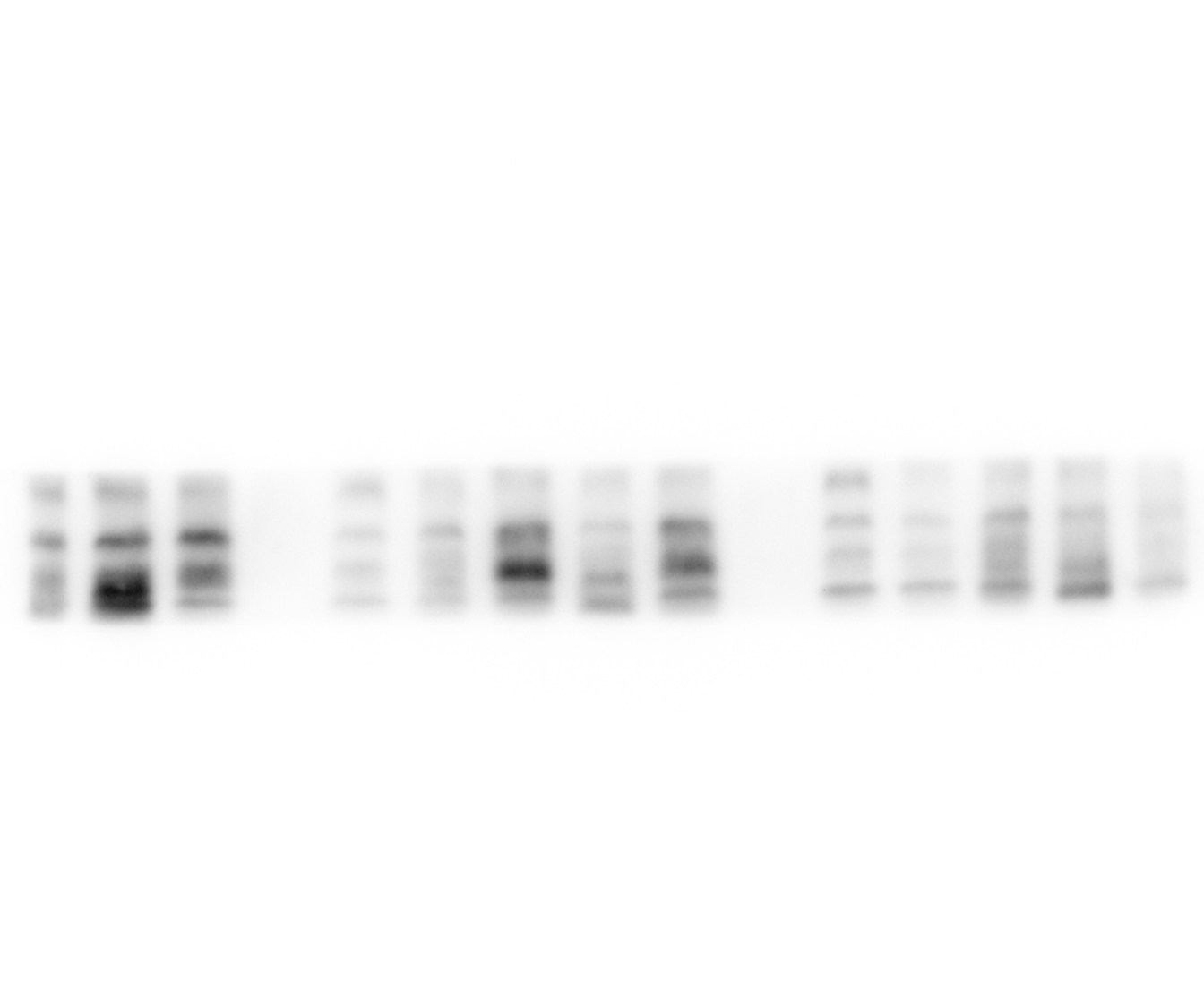

Supplement: Supplementary file 5 [file DataSheet_5.zip › 4D C-CASP3-6.Tif]

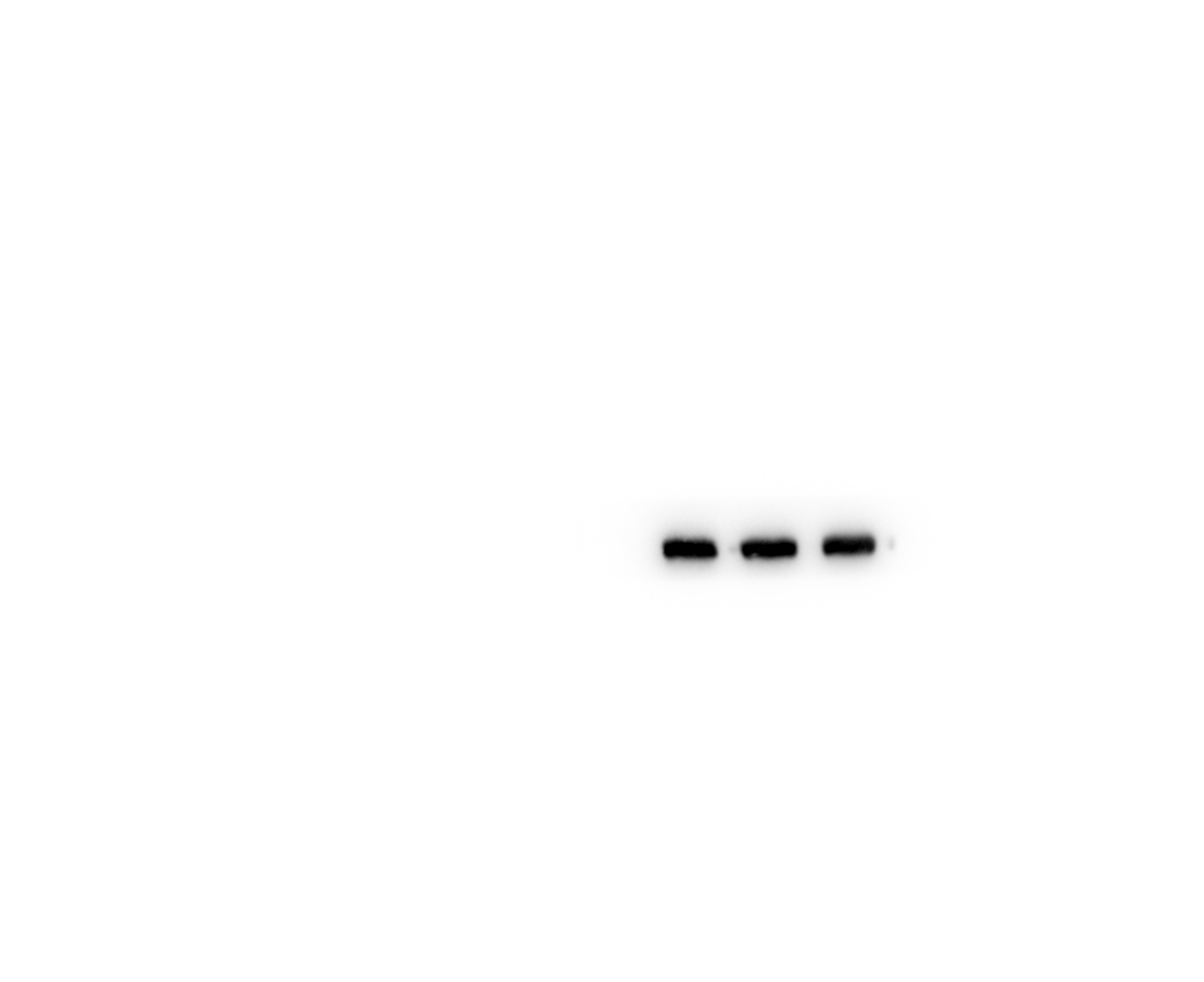

Supplement: Supplementary file 5 [file DataSheet_5.zip › 4D GAPDH-2 (2).Tif]

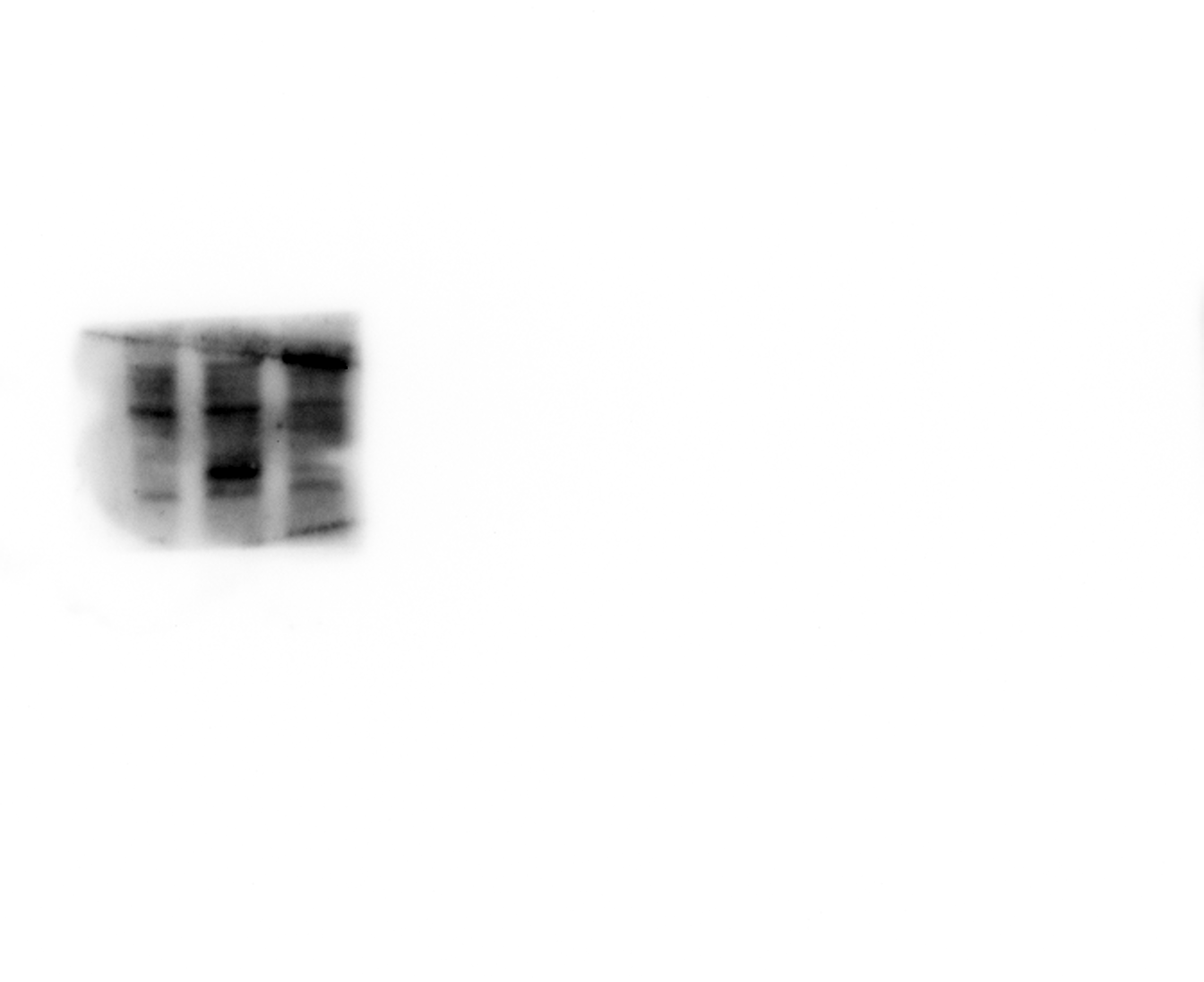

Supplement: Supplementary file 5 [file DataSheet_5.zip › 4D GSDME-2.Tif]
